# Single rhodium atoms anchored in micropores for efficient transformation of methane under mild conditions

**DOI:** 10.1038/s41467-018-03235-7

**Published:** 2018-03-26

**Authors:** Yu Tang, Yuting Li, Victor Fung, De-en Jiang, Weixin Huang, Shiran Zhang, Yasuhiro Iwasawa, Tomohiro Sakata, Luan Nguyen, Xiaoyan Zhang, Anatoly I. Frenkel, Franklin (Feng) Tao

**Affiliations:** 1 Department of Chemical and Petroleum Engineering and Department of Chemistry, University of Kansas, Lawrence, KS 66045 USA; 2 Department of Chemistry, University of California, Riverside, CA 92521 USA; 3 Department of Chemistry and Biochemistry, University of Notre Dame, Notre Dame, IN 46556 USA; 4 Innovation Research Center for Fuel Cells and Graduate School of Informatics and Engineering, The University of Electro-Communications, Chofu, Tokyo 182-8585 Japan; 5 State Key Laboratory of Photocatalysis on Energy and Environment and College of Chemistry, Fuzhou University, Fuzhou, 350116 China; 6 Department of Materials Science and Chemical Engineering, Stony Brook University, Stony Brook, NY 11794 USA; 7 Division of Chemistry, Brookhaven National Laboratory, Upton, NY 11973 USA

## Abstract

Catalytic transformation of CH_4_ under a mild condition is significant for efficient utilization of shale gas under the circumstance of switching raw materials of chemical industries to shale gas. Here, we report the transformation of CH_4_ to acetic acid and methanol through coupling of CH_4_, CO and O_2_ on single-site Rh_1_O_5_ anchored in microporous aluminosilicates in solution at ≤150 °C. The activity of these singly dispersed precious metal sites for production of organic oxygenates can reach about 0.10 acetic acid molecules on a Rh_1_O_5_ site per second at 150 °C with a selectivity of ~70% for production of acetic acid. It is higher than the activity of free Rh cations by >1000 times. Computational studies suggest that the first C–H bond of CH_4_ is activated by Rh_1_O_5_ anchored on the wall of micropores of ZSM-5; the formed CH_3_ then couples with CO and OH, to produce acetic acid over a low activation barrier.

## Abstract (web summary)

Catalytic transformation of CH_4_ under mild conditions has implications to shale gas utilization. Here, the authors report the transformation of CH_4_ to acetic acid through coupling of CH_4_, CO and O_2_ on single-site Rh_1_O_5_ anchored in microporous aluminosilicates in liquid phase.

## Introduction

CH_4_ has been one of the inexpensive energy resources since the maturation of hydraulic fracturing technology. So far, most processes of transformation of CH_4_ to intermediate compounds for chemical industries including steam or dry reforming, partial oxidation, and oxidative coupling are performed at high temperatures. One side effect of these processes is the deactivation of catalysts due to coke formation^1,2^. Another is the input of huge amount of energy since they are performed at high temperatures. Thus, activation of C–H of CH_4_ at a low temperature is necessary in order to transform shale gas to intermediate compounds of chemical industries in an energy-efficient manner^3–9^.

Acetic acid is one of the important intermediates of chemical industries. The global demand is 6.5 million metric tons per year (Mt/a). Currently, it is produced from methanol carbonylation, in which CO reacts with methanol to form acetic acid. However, methanol is synthesized from CO and H_2_, which are produced from steam reforming processes of either methane or coal at high temperatures^10^. Replacement of the current high-temperature catalysis toward production of acetic acid with catalysis at low temperatures would be feasible if a catalytic process on a heterogeneous catalyst could efficiently, directly transform CH_4_ to acetic acid under a mild condition. Transformations of methane to methanol and acetic acid on isolated palladium and rhodium atoms anchored in zeolite in liquid solution were simultaneously explored in our group since early 2012. We reported oxidation of methane to form methanol on Pd_1_O_4_ anchored in ZSM-5 in aqueous solution at low temperature in 2016^11^. Other than the mild oxidation of CH_4_ to CH_3_OH with supported isolated Pd atoms anchored in ZSM-5, we have simultaneously studied oxidization of methane through coupling with CO and O_2_ to methanol and acetic acid on isolated Rh atom anchored in ZSM-5 since 2012 and the Rh_1_O_5_/ZSM-5 catalyst was synthesized and its high activity was confirmed and its structure was identified before summer of 2014.

Formation of single sites is a significant approach to developing catalyst toward high catalytic activity and selectivity^11–17^. Separately anchoring catalytic sites (M_1_) on an oxide support (M_1_/A_x_O_y_) tunes the electronic state of catalytic sites of metal atoms (M_1_), which are typically continuously packed on surface of a metal nanoparticle or periodically located in a surface lattice of a metal oxide nanoparticle (M_x_O_y_). Compared to continuously packed M atoms on surface of a metal nanoparticle (····M-M-M····) and periodically packed M cations in surface lattice of a metal oxide nanoparticle (···O-M_1_-O-M_1_^*^-O-M_1_-O-M_1_-O ····), these isolated cation sites (M_1_^*^) anchored on surface of a substrate oxide (A_a_O_b_), ···O-A-O-M_1_^*^-O-A-O-A, exhibit a distinctly different coordination of M atoms. Thus, those isolated cations (M_1_) could exhibit a catalytic performance distinctly different from a metal oxide nanoparticle (M_x_O_y_) or a metal nanoparticle.

We separately anchored Rh cations on the internal surface of micropores of an aluminosilicate, H-ZSM-5 through ion exchange between Rh^3+^ in solution and H^+^ on the internal surface of micropore, similar to the isolated palladium atoms in ZSM-5^11^. As these Bronsted sites are typically isolated, Rh^3+^ cations can be separately anchored on Bronsted sites. Production of acetic acid through coupling CH_4_ with CO and O_2_ (2CH_4_+2CO+O_2_→2CH_3_COOH) is efficiently catalyzed by these singly dispersed Rh sites, Rh_1_O_5_. Different from rhodium atoms anchored on nonporous silicate and other nonporous oxide, this heterogeneous catalyst exhibits high activity in transforming CH_4_ to acetic acid, about 0.1 CH_3_COOH molecules per Rh site per second with a selectivity of ~70% for production of acetic acid. Isotope-labeled experiments using ^13^CH_4_ and ^13^CO show that CH_3_ of CH_3_COOH forms from activation of CH_4_ to CH_3_ and C=O of CH_3_COOH from insertion CO to Rh-OH. Density functional theory (DFT) calculation suggests that activation of C–H of CH_4_ and O–O of O_2_ are performed on a Rh_1_ atom and thus CH_3_- and HO are formed on the Rh_1_ atom. Insertion of C′O′ to Rh_1_–O bond of –Rh_1_–OH to form a –Rh_1_–C′O′OH on Rh_1_ atom; the formed –Rh_1_–C′O′OH couples with CH_3_ adsorbed on the same Rh_1_ atom, generating the first product molecule, CH_3_C′O′OH. The left –Rh_1_=O activates the second CH_4_ to form CH_3_ and HO;   the formed CH_3_ couples with CO to form acetyl, which couples with adsorbed HO to form the second acetic acid molecule.

## Results

### Preparation of isolated Rh catalytic site in ZSM-5

**Fig. 1:**
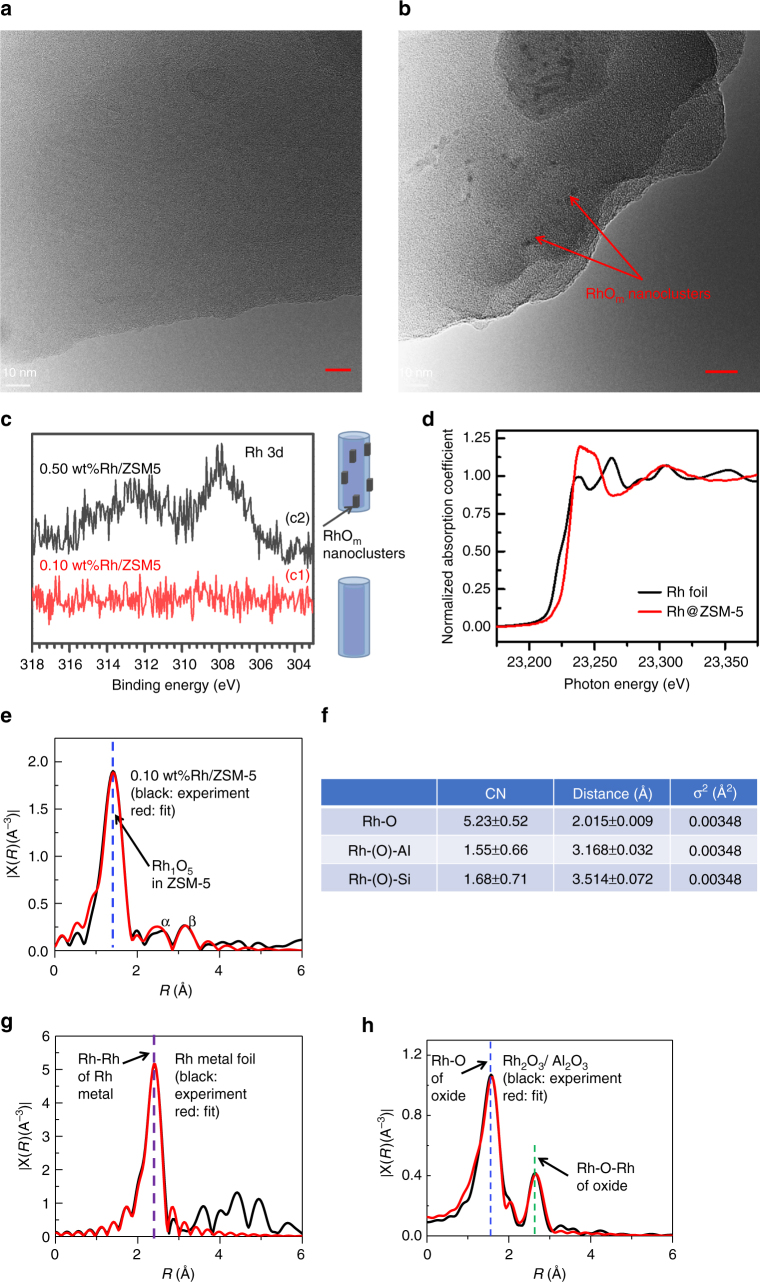
Structural characterization of isolated rhodium atoms in ZSM-5. **a** TEM image of particles of 0.10 wt%Rh/ZSM-5; scale bar: 10 nm. **b** TEM image of particles of 0.50 wt%Rh/ZSM-5; scale bar: 10 nm. **c** Rh 3d XPS peak of 0.l0 wt%Rh/ZSM-5 and 0.50 wt%Rh/ZSM-5. **d** Energy space of Rh K-edge of 0.l0 wt%Rh/ZSM-5 and Rh foil (reference sample) of X-ray absorption near edge spectra (XANES). **e** r-space of Rh K-edge of experimental (black) and calculated (red) data of the k^2^-weighted Rh K-edge EXAFS spectra of used 0.10 wt%Rh/ZSM-5. **f** Coordination number and bond length on average of the used 0.10 wt%Rh/ZSM-5. **g** r-space of Rh K-edge of experimental (black) and calculated (red) data of the k^2^-weighted Rh K-edge EXAFS spectra of Rh metal foil. **h** r-space of Rh K-edge of experimental (black) and calculated (red) data of the k^2^-weighted Rh K-edge EXAFS spectra of Rh_2_O_3_ nanoparticles supported on Al_2_O_3_

Rh cations were introduced to the internal surface of micropores of ZSM-5 through a method integrating vacuum pumping and incipient wetness impregnation (IWI). To minimize the amount of Rh cations to be deposited on external surface of a ZSM-5 particle, solution of Rh^3+^ with the same volume as the pore volume of ZSM-5 was slowly dropped to ZSM-5 powder with a syringe pump when the catalyst powder was continuously stirred and remained in vacuum. During IWI, Rh cations exchanged with singly dispersed Brønsted acid sites of H-ZSM-5, which was prepared through calcining NH_4_-ZSM-5 at 400 °C for 12 h. After the introduction of Rh^3+^, the samples were further dried in an oven at 80 °C for 3 h and calcined in air at 550 °C for 3 h, forming the catalyst, Rh/ZSM-5. The evolution of the chemical environment of Rh cations in ZSM-5 was shown in Supplementary Fig. 1. The concentration of Rh cations in the as-synthesized catalyst was measured through inductively coupled plasma atomic emission spectroscopy (ICP-AES). Before an ICP-AES measurement, 28 mg of 0.10 wt%Rh/ZSM-5 was dissolved in aqua regia. For catalyst with a nominal mass ratio of Rh to aliminosilicate, 0.10 wt%, the measured weight percent was 0.10 wt%, which suggests no obvious loss of Rh atoms during the preparation. X-ray photoelectron spectroscopy (XPS) studies of the as-synthesized 0.10 wt%Rh/ZSM-5 show the lack of Rh atoms in surface region of catalyst particles (red spectrum in Fig. 1c). The lack of Rh atoms in surface region revealed with XPS together with the 0.10 wt%Rh in the as-synthesized catalyst measured with ICP-AES suggests that these introduced Rh atoms were anchored in micropores of ZSM-5 particles instead of the external surface of ZSM-5 particles. At a high loading (0.50 wt%Rh/ZSM-5), unfortunately rhodium oxide nanoparticles (2–4 nm) were formed as evidenced by the low contrast patches in transmission electron microscopy (TEM) image (Fig. 1b), consistent with the observed Rh 3d photoemission feature in studies of sample using XPS (black spectrum in Fig. 1c)^18^.

The existence of Rh atoms in the microproes of ZSM-5 after catalysis was confirmed by the measured concentration of Rh atoms remained in micropores with ICP-AES, which was 0.098 %. Extended X-ray absorption fine structure spectroscopy (EXAFS) was used to characterize the chemical environment of anchored Rh atoms of used 0.10 wt%Rh/ZSM-5 (the catalyst after reaction). After catalysis, the used catalyst powder was centrifuged and thus washed with deionized H_2_O several times and then dried in oven at 200 °C. The obtained powder was used for EXAFS studies in flowing He at 150 °C. r-space spectrum of K-edge of Rh atoms of the used catalyst show that Rh atoms bond with oxygen atoms and the average coordination number of oxygen atoms to a Rh atom is CN(Rh-O) of 5.23 ± 0.52 (Fig. 1e, f). Notably, no contribution of Rh–Rh metal bonds was needed to fit the r-space spectrum of Rh K-edge (Fig. 1e), suggesting that there is no evidence for formation of Rh–Rh metal bonds. This is consistent with the oxidization state of Rh shown in Fig. 1h. Similar to literature^19–21^, the second shell of rhodium oxide at 2.60 Å in r-space spectrum (Fig. 1h) was clearly observed in our reference sample Rh_2_O_3_ nanoparticles. However, there is lack of Rh–O–Rh peak at 2.60 Å in the r-space spectrum of 0.10 wt%Rh/ZSM-5 (black line in Fig. 1e). It shows Rh atoms of our used catalyst do not have the second coordination shell of Rh atoms in terms of lack of Rh–O–Rh and thus there are no rhodium oxide nanoclusters formed in our used catalyst (0.10 wt%Rh/ZSM-5). Inspired by work of Gates group^14,22^, particularly the assignment of intensity at about 2.7 Å in r-space spectrum of Rh K-edge to Rh–O–Al with a Rh–(O)–Al distance of 3.02 Å^14,23–25^, we fit the small peak (α) at about 2.7 Å in Fig. 1e to Rh–O–Al. In addition, we fit the intensity at about 3.3 Å (β) in r-space of Rh K-edge to Rh–O–Si (Fig. 1e); the coordination numbers of Al to Rh through O atom and Si to Rh through O atom are 1.55 ± 0.66 and 1.68 ± 0.71, respectively; the distances of Al and Si atoms to the Rh atoms are 3.168 ± 0.032 Å and 3.514 ± 0.072 Å, respectively. Thus, these EXAFS studies show that Rh atoms of 0.10 wt%Rh/ZSM-5 are singly dispersed in micropores of ZSM-5 and each Rh atom bond with about five oxygen atoms on average. In the following paragraphs, sometimes we used Rh_1_O_5_@ZSM-5 when we need to point out the coordination environment of the Rh atoms on average. Supplementary Fig. 1f schematically shows the structure of a catalytic site of Rh_1_O_5_ anchored in micropores of ZSM-5.

The replacement of Brønsted acid sites (BAS) of H-ZSM-5 by Rh cations was confirmed with ^1^H NMR (nuclear magnetic resonance) of 0.10 wt%Rh/ZSM-5 and H-ZSM-5. As shown in Supplementary Fig. 2a, the peak at 4.6 ppm was assigned to BAS site of H-ZSM-5 (Supplementary Table 1)^26,27^. It was not obviously observed in the same region of chemical shift in Supplementary Fig. 2b. This difference in Supplementary Fig. 2 suggests that the loss of some BAS sites due to ion exchange of Rh^3+^ with H^+^ in the IWI process.

### Catalytic performance of Rh_1_O_5_@ZSM-5 at 150 °C

Catalytic activities of pure H-ZSM-5 and as-prepared Rh/ZSM-5 catalysts were measured by adding 28 mg catalyst to 10 mL deionized water in a Parr high-pressure reactor (Supplementary Fig. 3b). The aqueous solution with dispersed catalyst particles was continuously, vigorously stirred by a magnetic bar coated with plastic materials at a speed of 600 rpm during catalysis. A mixture of CH_4_, CO, and O_2_ with different partial pressure was introduced to the Parr reactor at room temperature. A portion of these reactant gases with a relatively high-pressure can diffuse to micropores of catalyst dispersed in the solvent (H_2_O or dodecane) and thus be catalyzed. Then, the reactor was heated to a set temperature. The reaction temperature of the solvent was directly measured through a thermocouple probe submerged to the solution consisting of the dispersed catalyst particles and solvent in the Parr reactor. The preservation of catalysis temperature for certain amount of time were performed by a temperature controller. This chemical transformation was performed for certain amount of time. The pressure, reaction temperature, and reaction time of each measurement of catalytic performance were given in the following figures and tables. Catalytic reaction under each condition was repeated at least four times.

After each catalytic reaction, the solution in the Parr reactor consisting the used catalyst powder and liquid products was filtrated to separate the used catalyst powder. The clear liquid obtained after filtering the catalyst powder mainly contains acetic acid, methanol, formic acid, and solvent. The product solution was analyzed by ^1^H NMR and ^13^C NMR. The measurement was calibrated with 3-(trimethylsilyl)-1-propanesulfonic acid sodium salt (DSS) with chemical shift at *δ* = 0.0 ppm^11,28^. A DSS solution was prepared by dissolving DSS to D_2_O, making a solution with concentration of DSS in D_2_O at 0.020 wt%. Typically, 0.70 mL of the obtained clear liquid solution was mixed with 0.10 mL of as-prepared DSS solution in an NMR tube before NMR analysis. The identified oxygenate products were acetic acid (*δ* = 2.08 ppm), formic acid (*δ* = 8.28 ppm) and methanol (*δ* = 3.33 ppm). A solvent suppression program was applied for minimizing the signal originating from H_2_O, similar to our previous studies^11,28^. Supplementary Fig. 4 is a representative NMR spectrum of solution after catalysis; peak of DSS was marked on it. To quantify the amounts of products, standard curves of acetic acid, formic acid, and methanol were carefully established and shown in Supplementary Fig. 5. The analysis was described in the Methods section.

**Fig. 2:**
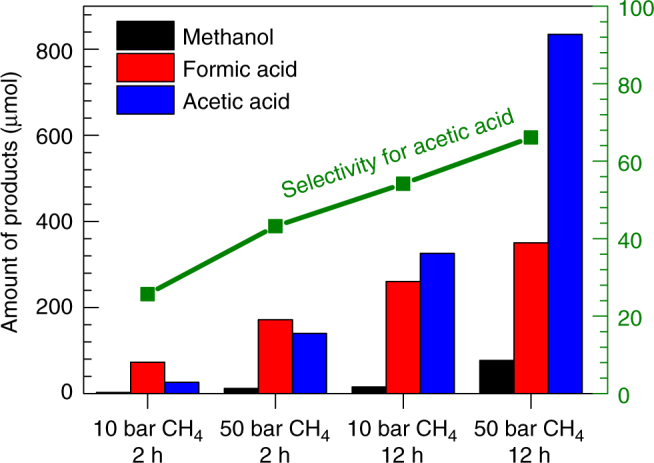
Catalytic performance of 0.10 wt%Rh/ZSM-5. Yields of acetic acid, formic acid and methanol as well as the selectivity to acetic acid as a function of different pressures of CH_4_ (10 or 50 bars) and different reaction times (2 h or 12 h). 28 mg  of 0.10 wt%Rh/ZSM-5 was used for each catalysis test. Each catalysis test used 10 bar CO and 8 bar O_2_ and certain pressure of CH_4_ as noted on *x*-axis (10 or 50 bars). The catalysis temperatures of all studies here were 150 °C

With 28 mg of 0.10 wt%Rh/ZSM-5, 226.1   μmol of total products (acetic acid, formic acid, and methanol) were produced at 150 °C in the first hour (entry 4 in Supplementary Table 2). Under the same condition, unfortunately the yields of the total organic compounds formed from 0.50 wt%Rh/ZSM-5 (entry 5 in Supplementary Table 2) are similar to 0.10 wt%Rh/ZSM-5. The similarity in catalytic performances of the two catalysts shows that the rhodium oxide nanoparticles formed on the surface of 0.50 wt%Rh/ZSM-5 are not active for this transformation. In other words, the catalytic activity of 0.10 wt%Rh/ZSM-5 in the production of acetic acid was contributed from the Rh_1_O_5_ sites anchored in micropores of ZSM-5 instead of rhodium oxide nanoparticles supported on the external surface of ZSM-5. As the conversions of CH_4_ in these studies of Fig. 2 and Table 1 are lower than 20%, we used these conversions and yields to calculate the turn-over rates with the equation:

1 $${\mathrm{TOR}} = \frac{{{\mathrm{Number}}\;{\mathrm{of}}\;{\mathrm{produced}}\;{\mathrm{moelcules}}}}{{\mathrm{Time}}\;{\mathrm{of}}\;{\mathrm{catalytic}}\;{\mathrm{reaction}}\;( {\mathrm{S}} ) \times {\mathrm{Number}}\;{\mathrm{of}}\;{\mathrm{acitve}}\;{\mathrm{sites}}\; ( {{\mathrm{Rh}}_1{\mathrm{O}}_5} )}$$

**Table 1:** Comparison of TOR for formation of acetic acid or oxygenates including acetic acid, formic acid and methanol on 0.10 wt%Rh/ZSM-5

Entry	Catalyst	Catalytic temperature	TOR of acetic acid (molecule per site per second)	TOR of organic oxygenate^**a**^ (number of molecules per site per second)	Selectivity for production of acetic acid
1	0.10 wt%Rh/ZSM-5^a^	150 °C	0.040^b^	0.099^b^	40.0%
2	0.10 wt%Rh/ZSM-5^a^	150 °C	0.070^c^	0.10^c^	70.1%
3	Rh(NO_3_)_3_^a^	150 °C	6.3 ×10^-6 d^	2.4×10^-5 d^	26.3%

^a^ Here the organic oxygenates include acetic acid, formic acid and methanol (CO_2_ was not included). Calculations of TORs of these catalysts were described in Supplementary Methods

^b^ The catalysis condition of 0.10  wt%Rh/ZSM-5: mixture of 50  bar CH_4_, 10  bar CO, 8  bar O_2_, 2  h; the yields of acetic acid and all organic oxygenates were plotted in Fig. 2

^c^ The catalysis condition of 0.10  wt%Rh/ZSM-5: mixture of 50  bar CH_4_, 10  bar CO, 8  bar O_2_, 12  h; the yields of acetic acid and all organic oxygenates were listed in Fig. 2

^d^ Rh(NO_3_)_3_ was used in literature^32^. Five milliliters of 0.01 mol/L Rh(NO_3_)_3_ was added in Parr reactor and 50  bar CH_4_, 10  bar CO, and 8  bar O_2_ were introduced to the Parr reactor and then the Parr reaction was sealed; the reaction was performed at 150  °C for about 90  h. This measurement was done for comparison with 0.10  wt%Rh/ZSM-5 in which Rh_1_ cations were anchored in micropores

This calculation is based on an assumption that all loaded Rh atoms are active sites. This calculation was further described in Supplementary Methods. The activities for production of acetic acid and total organic oxygenates including acetic acid, methanol and formic acid at 150 °C are 0.070 and 0.10 molecules per Rh atom per second (entry 2 of Table 1), respectively.

To check whether Rh atoms anchored in micropores of ZSM-5 could detach from ZSM-5, the clear solution was obtained by filtration for removal of Rh/ZSM-5 catalyst particles from the solution after catalysis at 150 °C for 12 h. ICP-AES test of this solution shows that only 2% of the total Rh atoms of 28 mg of 0.10 wt%Rh/ZSM-5 detached from ZSM-5 to solution. Thus, most Rh atoms remained in ZSM-5 after catalysis. Due to the negligible amount of Rh^3+^detached from 0.10 wt%Rh/ZSM-5 and the extremely low TOF of free Rh^3+^ in solution evidenced in entry 3 in Table 1, contribution of the detached Rh^3+^ to the measured catalytic activity in formation of acetic acid is negligible. It suggests that the anchored Rh atoms are the active sites.

To further confirm the contribution of Rh_1_O_5_ sites to the formation of acetic acid, control experiments were performed on these catalysts including 28 mg of H-ZSM-5, 28 mg of 0.10 wt%Rh/SiO_2_ and 28 mg of 0.10 wt%Rh/Al_2_O_3_ under the exactly same condition as that of 28 mg 0.10 wt%Rh/ZSM-5 at 150 °C in the mixture of 10 bar CH_4_, 10 bar CO and 8 bar O_2_ for 4 h. As shown in Table 2, the amounts of acetic acid, formic acid, or methanol produced on 28 mg of 0.10 wt%Rh/SiO_2_ and 28 mg of 0.10 wt%Rh/Al_2_O_3_ are lower than 10 μmol, which are at the level of error bar. All the reported yields in this communication are the measured products formed from 28 mg catalyst. The yield could be shown as μmol/gram catalyst by multiplying a factor of$\frac{{1000\;{\rm mg}/{\rm gram}}}{{28\;{\rm mg}}}$. For example, the measured yields of methanol and acetic acid on 28 mg of 0.10%Rh/SiO_2_ are 8.70 and 6.13 μmol, respectively; if they are multiplied by the factor $\frac{{1000\;{\rm mg}/{\rm gram}}}{{28\;{\rm mg}}}$ they seem to indicate that 310 μmol methanol and 218 μmol acetic acid could form from one gram of 0.10%Rh/SiO_2_ catalyst. Here, the multiplication is not meaningful since the values in Table 2 are at the uncertainty level. As the measured 8.70 μmol methanol and 6.13 μmol acetic acid from 28 mg 0.10%Rh/SiO_2_ catalyst are in the range of error bar of these measurements, these values are not used to predict activity of 1 gram to compare with other catalysts. Even if the multiplication factor were applied, the activity of 0.10%Rh/SiO_2_ is significantly lower than 0.10 wt%Rh/ZSM-5. For instance, 218 μmol acetic acid from per gram 0.10%Rh/SiO_2_ calculated from the measured 6.13 μmol acetic acid per 28 mg is still much lower than 5000 μmol acetic acid from per gram 0.10 wt%Rh/ZSM-5 calculated from the measured 140 μmol acetic acid per 28 mg catalyst. In conclusion, these control samples in terms of Rh supported on these nonporous oxides and even on a couple of commonly used reducible oxides are not active for the production of acetic acid or methanol from coupling of CH_4_ with CO and O_2_. Thus, these studies suggest the significant contribution of Rh_1_O_5_ sites encapsulated in ZSM-5 to the formation of acetic acid.

**Table 2:** Catalytic performances of 28 mg catalysts of 0.10 wt%Rh supported on different supports in a mixture of 10 bar CH_4_, 10 bar CO, and 8 bar O_2_ at 150 °C for 3 h with 10 mL H_2_O in a high-pressure reactor

Entry	Samples	Methanol (μ mol)	Formic acid (μ mol)	Acetic acid (μ mol)	Total products (μ mol)
1	H-ZSM-5	3.67	2.28	1.87	7.82
2	0.10%Rh/SiO_2_	8.70	4.62	6.13	19.46
3	0.10%Rh/Al_2_O_3_	5.68	0.91	3.05	9.64

Acetic acid, formic acid, and methanol were identified as products. Reactants on pure H-ZSM-5 was also performed at the same conditions as blank experiment (entry 1)

The participation of all these three reactants (CH_4_, CO, and O_2_) was confirmed with three parallel studies on 0.10 wt%Rh/ZSM-5 under the exactly same catalytic condition as listed in Supplementary Fig. 6a, b, c; in each of these studies, only two of the three reactants were introduced to the Parr reactor; none of these studies produced acetic acid, formic acid, or methanol due to the lack of the third reactant gas. Those studies clearly show that all the three gases (CH_4_, CO, and O_2_) are necessary reactants for the formation of CH_3_COOH. The necessity of the three reactants was supported by DFT calculations described later.

### Participation of molecular O_2_ in synthesis of acetic acid

**Fig. 3:**
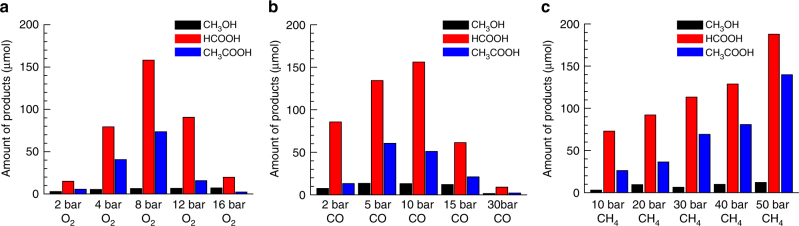
Influence of partial pressure O_2_, CO and CH_4_ on catalytic performances. Yields of methanol (black), formic acid (blue), and acetic acid (red) in the chemical transformation of CH_4_ at 150 °C in aqueous solutions at different pressure of O_2_, CO, CH_4_. **a** 35 bar CH_4_, 10 bar CO, and different pressure of O_2_ at 150 °C for 2 h. **b** 50 bar CH_4_, 8 bar O_2_, and different pressure of CO at 150 °C for 1.5 h. **c** 10 bar CO, 8 bar O_2_ and different pressure of CH_4_ at 150 °C for 2 h

Here, we used low-cost molecular oxygen (O_2_) or compressed air as oxidant in oxidative transformation of CH_4_ and CO to acetic acid. To further confirm the direct participation of molecular O_2_, we performed catalysis at different pressures of O_2_ (2, 4, 8, 12, and 16 bar) but all other conditions are the same in these parallel studies; in each of these parallel studies, 28 mg of 0.10 wt%Rh/ZSM-5 was added to 10 mL H_2_O. The reaction was performed at 150 °C for 2 h in a mixture of 35 bar CH_4_, 10 bar CO and different pressures of O_2_, in order to investigate the correlation of yields of products (acetic acid, formic acid, and methanol) with pressure of O_2_. As shown in Fig. 3a, highest yields of acetic acid and formic acid were obtained from the catalysis using 8 bar O_2_. The increase of yield of acetic acid and formic acid along the increase of O_2_ pressure shows that O_2_ does participate in the formation of acetic acid and formic acid. It is expected that high coverage of oxygen atoms on a Rh atom achieved with high-pressure O_2_ could saturate Rh_1_ atom with oxygen atoms, which poisons catalytic sites and thus results in a low yield of oxygenates at high pressure of O_2_.

### Direct participation of CO to synthesis of acetic acid

To further confirm the participation of CO in the formation of acetic acid, influence of the partial pressure of CO on both the conversion of CH_4_ and selectivity for production of acetic acid was investigated through parallel studies (Fig. 3b). In each of these studies, the partial pressures of CH_4_ and O_2_ were fixed at 50 and 8 bar, respectively. However, the pressures of CO in the five studies are 2, 5, 10, 15, and 30 bar. The increase of the amount of acetic acid while CO pressure was increased from 2 to 10 bar suggests that CO directly participates into the formation of acetic acid. However, the lack of activity for production of acetic acid at 30 bar CO showed that catalyst sites were blocked at such a high-pressure of CO. Clearly, CO molecules must have directly interacted with the Rh cations. At high-pressure of CO, high coverage of CO could saturate the coordination of a Rh_1_ atom and thus deactivate this catalyst. We measured the concentrations of Rh in the liquid (α) after filtration of the catalyst experienced the catalysis at 10 bar CO, 50 bar CH_4_, and 8 bar O_2_ for 2.5 h, and in another liquid (β) after filtration of the catalyst experienced the catalysis at 30 bar CO, 50 bar CH_4,_ and 8 bar O_2_. The amounts of Rh atoms in the liquids α and β are 2.0% and 13.0% of all Rh atoms of 28 mg of 0.10 wt%Rh/ZSM-5, respectively. Thus, the much larger loss of Rh atoms at high-pressure of CO (30 bar) suggests that Rh atoms formed carbonyl in CO at high-pressure and some of these formed rhodium carbonyl species desorbed from micropores and then dissolved in the solution. Thus, some Rh species detached at high pressure of CO.

**Fig. 4:**
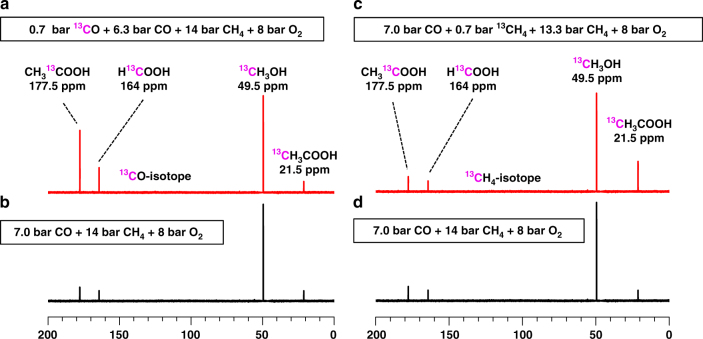
^13^C NMR studies of reaction using ^13^CO or ^13^CH_4_. ^13^C NMR spectra of products of acetic acid, formic acid, and methanol on 28 mg 0.10 wt%Rh/ZSM-5 at 170 °C for 10 h in gas of **a** mixture of 0.7 bar ^13^CO, 6.3 bar CO, 14 bar CH_4,_ and 8 bar O_2_, **b** mixture of 7 bar CO, 14 bar CH_4_, and 8 bar O_2_, **c** mixture of 7 bar CO, 0.7 bar ^13^CH_4_, 13.3 bar CH_4_, and 8 bar O_2_, and **d** mixture of 7 bar CO, 14 bar CH_4_, and 8 bar O_2_. **a** and **b** are isotope experiments; **c** and **d** are their corresponding contrast experiments

A molecular-level evidence on direct participation of CO in the synthesis of acetic acid is the following isotope experiment. 0.7 bar ^13^CO (Aldrich, 99%, total pressure 2.5 bar) was mixed with 6.3 bar of CO, 14 bar CH_4,_ and 8 bar O_2_ for catalysis of 10 h (Fig. 4a). As the chemical shift of ^13^CH_3_OH in ^13^C spectrum can be readily distinguished from acetic acid and formic acid, 40 μmol of ^13^CH_3_OH (Aldrich, 99 at%) was added to the collected solution after catalysis as a reference to quantify the amount of potential isotope products ^13^CH_3_COOH, CH_3_^13^COOH, or H^13^COOH. As the unlabeled CO gas has a natural abundance of ^13^C of 1.10%, a small amount of CH_3_^13^COOH or H^13^COOH can form from the natural 1.10% ^13^CO of unlabeled CO gas tank. Contrast experiments using the mixture of 7 bar of CO, 14 bar CH_4_, and 8 bar O_2_ were performed (Fig. 4b). The intensity ratio of the formed CH_3_^13^COOH to ^13^CH_3_OH in the solution of isotope experiment using ^13^CO (Fig. 4a) is obviously larger than those formed in the contrast experiment using unlabeled CO (Fig. 4b) by 6.4 times; in addition, the intensity ratio of H^13^COOH to ^13^CH_3_OH in isotope experiment (Fig. 4a) is higher than that in the contrast experiment by 2.6 times (Fig. 4b). They suggested that the C′ atoms of CH_3_C′OOH and HC′OOH came from C′O molecules. Notably, the intensity ratio of ^13^CH_3_COOH to reference (^13^CH_3_OH) in isotope experiment (Fig. 4a) is the same as the ratio of the contrast experiment (Fig. 4b). It suggests that the C atoms of CH_3_ of CH_3_COOH do not come from the reactant CO.

One potential pathway to form acetic acid is the coupling of CO with a formed formic acid molecule; if so, yield of acetic acid should increase along the increase of CO pressure. However, as shown in Fig. 3b yield of acetic acid decreases along with the increase of CO pressure (≥10 bar). Thus, coupling formic acid with CO to form acetic acid is not a pathway. To further check the possibility of reaction between HCOOH and CO to form acetic acid, we performed three control experiments at 150 °C for 3 h under the following conditions including mixture of 28 mg 0.10 wt%Rh/ZSM-5, 108 μmol HCOOH, and 10 mL DI H_2_O without any CO, mixture of 28 mg 0.10 wt%Rh/ZSM-5, 108 μmol HCOOH, and 10 mL DI H_2_O with 5 bar CO, and mixture of 28 mg 0.10 wt%Rh/ZSM-5, 108 μmol HCOOH, and 10 mL DI H_2_O with 10 bar CO. As shown in Supplementary Fig. 7, no acetic acid was formed in these experiments.

### Direct participation of CH_4_ in formation of CH_3_COOH

The influence of CH_4_ pressure on the catalytic performance was explored at 150 °C under a mixture of 10 bar CO and 8 bar O_2_ and different pressure of CH_4_ (10, 20, 30, 40, and 50 bar) for 2 h (Fig. 3c). The progressive increase of yield of acetic acid along the increase of CH_4_ pressure shows that CH_4_ directly participates into the formation of acetic acid (Fig. 3c), which excluded a pathway in which CH_4_ couples with formic acid to form acetic acid. If acetic acid were formed from a coupling of formic acid with CH_4_, the amount of formic acid should have decreased along the increase of pressure of CH_4_ since more formic acid should have been consumed along with the increased amount of CH_4_.

To elucidate the source of carbon atoms at the molecular level, ^13^CH_4_ isotope experiments were performed. 0.7 bar ^13^CH_4_ (Aldrich, 99 at%) was mixed with 13.3 bar of CH_4_, 7.0 bar CO, and 8.0 bar O_2_ for isotope experiment on 28 mg of 0.10 wt%Rh/ZSM-5 at 170 °C for 10 h (Fig. 4c). A control experiment using 14 bar unlabeled CH_4_ was performed under the exactly same catalytic condition (Fig. 4d). ^13^CH_3_COOH were formed in the two experiments. However, their ratio of ^13^CH_3_COOH to reference (^13^CH_3_OH) when ^13^CH_4_ was used (Fig. 4c), is much larger than that when unlabeled CH_4_ was used (Fig. 4d). This difference shows that the carbon atom of CH_3_ of acetic acid comes from CH_4_ instead of CO. If C atoms of C=O of CH_3_COOH could come from CH_4_, the ratio of CH_3_^13^COOH to reference (^13^CH_3_OH) in Fig. 4c would be much larger than the ratio in Fig. 4d since the experiment of Fig. 4d contains significant amount of ^13^CH_4_. In fact, in both experiments (Fig. 4c, d), we did observe small amount of CH_3_^13^COOH but there is no difference between their ratios to reference (^13^CH_3_OH) in the experiments of both Fig. 4c, d. Here, the formation of CH_3_^13^COOH is due to the natural abundance of ^13^CO in unlabeled CO. Thus, CO does not contribute to the formation of CH_3_ of CH_3_COOH.

### Direct coupling of reactants for formation of acetic acid

**Fig. 5:**
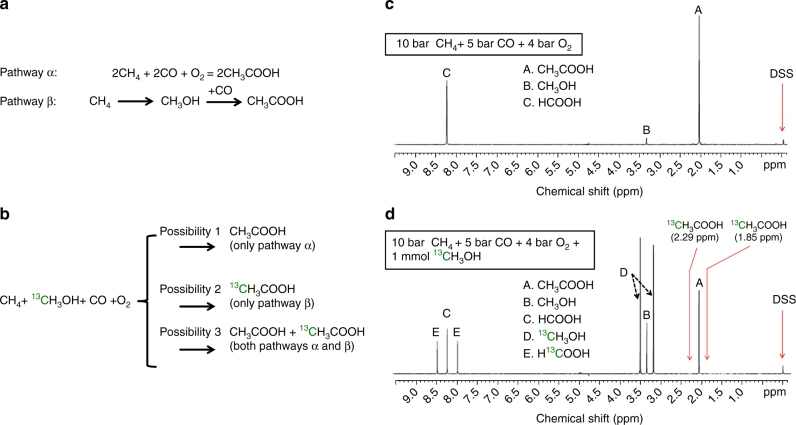
Isotope studies for elucidating whether acetic acid could be formed through coupling methanol with CO. **a** Two potential pathways α and β for production of acetic acid; in pathway α, CH_3_OH is not an intermediate compound for formation of CH_3_COOH; in pathway β, CH_3_OH is an intermediate compound  for formation of CH_3_COOH. **b** Potential catalytic products formed from 0.10 wt%Rh/ZSM-5 in the mixture of ^13^CH_3_OH and H_2_O in solution under mixture of CH_4_, CO, and O_2_ if the transformation of CH_4_, CO, and O_2_ follows pathway α, β, or both α and β. **c** NMR spectra of the products formed from 28 mg of 0.10 wt%Rh/ZSM-5 after reaction in 10 bar CH_4_, 5 bar CO, and 4 bar O_2_ at 150 °C for 1 h; there was no any isotope-labeled methanol, ^13^CH_3_OH added to the rector before this catalysis test. **d** NMR spectra of the products formed from 28 mg of 0.10 wt%Rh/ZSM-5 after reaction in 10 bar CH_4_, 5 bar CO, and 4 bar O_2_ at 150 °C for 1 h.; notably, 1.0 mmol ^13^CH_3_OH was added to H_2_O before this catalysis test

It is noted that the amounts of the observed methanol in any of our studies of this work are always much lower than acetic acid and formic acid. One potential argument for the low yield of methanol could be that methanol has been formed but it could have acted as an intermediate compound in the formation of acetic acid; in other words, formic acid  could have been consumed through coupling with CO to form acetic acid. Depending on whether CH_3_OH could act as an intermediate product in the formation of acetic acid or not, two categories of potential pathways α and β were proposed in  Fig. 5a. In potential pathway α, CH_4_ couples with CO to directly form acetic acid; methanol is not an intermediate product of this type of reaction pathway. In potential pathway β, however, CH_3_OH is an intermediate product and then be consumed in the formation of acetic acid; CH_4_ is first oxidized to CH_3_OH (the first step) and then CH_3_OH couples with CO to form acetic acid (the second step); the second step of the potential pathway β is called carbonylation of methanol by CO; it is in fact the Monsanto process^29–31^. In order to identify whether CH_3_OH carbonylation (pathway β) could be a pathway for the production of acetic acid on our catalyst Rh_1_O_5_@ZSM-5, carefully designed isotope experiments described in Supplementary Methods were performed.

These isotope experiments show that acetic acid cannot be formed from carbonylation of methanol by CO on our catalyst. In one isotope-labeled experiment, 1.0 mmol isotope-labeled ^13^CH_3_OH (99 atom% ^13^C, Aldrich) was added to 10 mL deionized H_2_O before introduction of 10 bar CH_4_, 5 bar CO, and 4 bar O_2_ to the Parr reactor. If CH_3_OH could not be an intermediate for formation of acetic acid, the added ^13^CH_3_OH would not particulate into the formation of isotope-labeled acetic acid, ^13^CH_3_COOH. Thus, no ^13^CH_3_COOH could be observed if carbonylation of methanol by CO would not be involved (possibility 1 in Fig. 5b). The NMR spectrum of the solution of products formed in the reactor having ^12^CH_4_, ^12^CO, and O_2_ in H_2_O (^13^CH_3_OH was not added) after reaction of 2 h was presented in Fig. 5c. Figure 5d is the NMR spectrum of the products formed after the catalysis for 1 h under a condition of mixture of ^13^CH_3_OH, ^12^CH_4_, ^12^CO, and O_2_ at 150 °C. The observed peaks A, B, C, D, and E in Fig. 5d were assigned to CH_3_COOH, CH_3_OH, HCOOH, ^13^CH_3_OH, and H^13^COOH, respectively. As neither peak of H atoms of ^13^CH_3_ of ^13^CH_3_COOH in ^1^H spectrum nor peak of ^13^C atoms of CH_3_^13^COOH in ^13^C spectrum was observed in the NMR, pathway β is not a pathway for formation of acetic acid. Thus, these isotope studies showed that acetic acid is not formed from carbonylation of methanol by CO. Additionally, H^13^COOH was observed clearly in Fig. 5d, suggesting that ^13^CH_3_OH can be oxidized to H^13^COOH under the current catalytic condition.

We also performed the dry reforming of CH_4_ by CO_2_ by introducing 30 bar CH_4_ and 30 bar CO_2_ to the reactor containing 10 mL H_2_O and well dispersed 28 mg of 0.10 wt%Rh/ZSM-5. The reactor was heated to 150 °C and remained at 150 °C for 5 h and then cooled to 10 °C in ice water. As shown in Supplementary Fig. 6d, NMR test shows none of these products (acetic acid, formic acid, and methanol) was formed.

### Ready separation of products from hydrophobic solvent

The above chemical transformation was performed in aqueous solution. As the products of this chemical transformation, acetic acid, formic acid, and methanol are hydrophilic, it is not readily to separate these hydrophilic products from water. To make these hydrophilic products automatically separate from solvent after synthesis, a hydrophobic solvent, *n*-dodecane was used. As shown in Supplementary Fig. 8 28 mg 0.10 wt%Rh/ZSM-5 in 10 mL *n*-dodecane in the mixture of 30 bar CH_4_, 10 bar CO, and 5 bar O_2_ is definitely active for the formation of acetic acid. The significant advantage of using the hydrophobic solvent is that the hydrophilic products of this reaction including acetic acid, methanol, and formic acid can be readily separated from the hydrophobic solvent, without or with a low energy cost.

### Feature of this mild oxidation of methane in solution

CH_4_ and CO can be oxidized with different oxidants including O_2_, concentrated H_2_SO_4_, or a superacid^32–34^ by using homogeneous catalyst^32^, in which acetic acid and other products (formic acid and methanol) were formed. One control experiment using Rh(NO_3_)_3_ was done (entry 3 in Table 1); the turn-over-rate (TOR) of the homogenous catalyst, Rh(NO_3_)_3_ without a promoter is only 6.3 × 10^−6^ molecules per rhodium cation per second at 150 °C. Here, the Rh_1_O_5_@ZSM-5 catalyzes the oxidation of CH_4_ and CO with a low-cost oxidant, molecular oxygen or even air at 150 °C at a solid–liquid–gas interface. TOR of the catalytic sites Rh_1_O_5_ anchored in microporous silicate reaches 0.070 CH_3_COOH molecules on per Rh_1_O_5_ site per second in mixture of 50 bar CH_4_, 10 bar CO, and 8 bar O_2_ (entry 2 of Table 1). These TORs for production of acetic acid on singly dispersed site Rh_1_O_5_ are much higher than those reported homogenous catalysts^32,33^ by >1000 times. As shown in Fig. 2, 840 μmol of acetic acid, 352 μmol of formic acid, and 82 μmol of methanol were produced from 28 mg 0.10 wt%Rh/ZSM-5 at 150 °C for 12 h under a catalytic condition of 50 bar CH_4_, 10 bar CO and 8 bar O_2_, which correspond to conversion of 10.2% of CH_4_ under this condition. Selectivity for production of acetic acid among all organic products reaches about 70% under this condition. Other than the highest catalytic efficiency on Rh_1_O_5_@ZSM-5, a significant advantage of our catalytic process is the ready separation of liquid products from the solid catalyst and solvent.

It is found that a shorter reaction time gives a higher selectivity for formation of formic acid and a longer reaction time lead to a higher selectivity for formation of acetic acid. As shown in Supplementary Fig. 9 both formic acid and acetic acid are the main products when reaction time is shorter than 3 h. When the reaction time is 3 h or longer, acetic acid is the main product. The evolution of the yields of formic acid and acetic acid as a function of time implies that the relative low temperature of catalyst in the heating from 25 to 150 °C is favorable for the formation of formic acid. More discussion on time-dependent selectivity for formation of formic acid and acetic acid can be found from Supplementary Discussion.

### Understanding reaction mechanism at molecular level

**Fig. 6:**
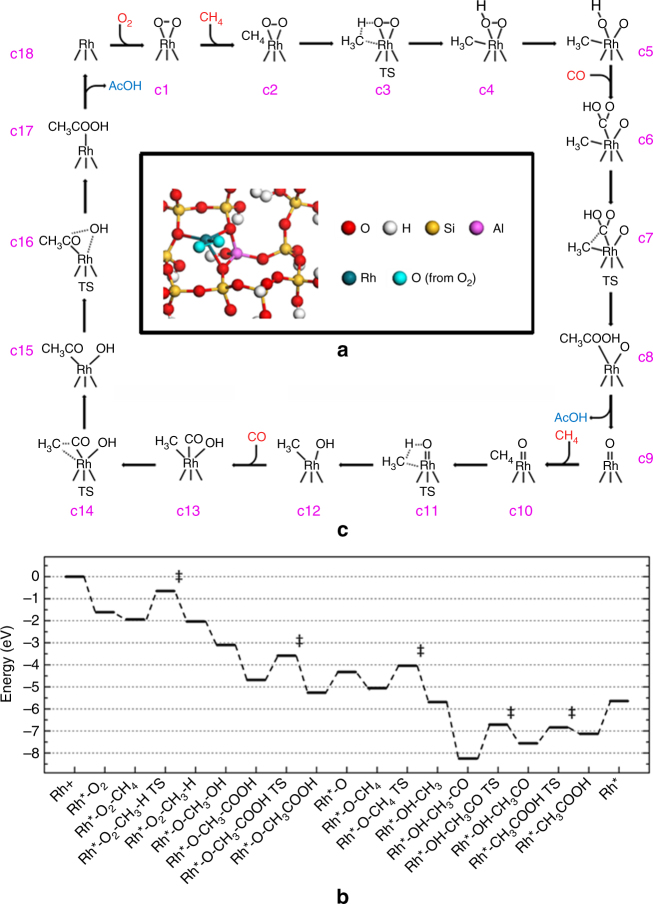
Computational studies of reaction pathway. Minimum-energy paths and reaction schematic for formation of acetic acid from CH_4_, CO, and O_2_ on Rh_1_O_5_/ZSM-5. The formation of acetic acid is illustrated in a catalytic cycle starting with the singly dispersed Rh_1_O_5_ site. The balanced reaction cycle consumes one O_2_, two CH_4_, and two CO to make two CH_3_COOH molecules (2CH_4_+2CO+O_2_=2CH_3_COOH). **a** The optimized catalytic sites, Rh_1_O_5_ anchored on Brønsted site in microspore of ZSM-5. **b** Energy profile for pathway of transforming CH_4_, CO, and O_2_ to CH_3_COOH. **c** Intermediates and transition states for a complete catalytic cycle, starting with Rh_1_O_5_ (c1). Transition states are highlighted with the double dagger symbols

Based on the coordination environment of Rh_1_ atoms suggested by EXAFS studies, we used a structural model whose Rh atom bonds with three oxygen atoms of the substrate wall and two oxygen atoms of one oxygen molecule in our computational studies. Our DFT calculations suggest that the Rh atom prefers a ten-membered-ring channel, which has smaller repulsion, instead of a six-membered ring channel of ZSM-5. Based on the experimental preparation method, we expect that the Rh_1_ cations replace the Bronsted sites and thus bind to the Al atoms. As shown in Fig. 6a this Rh_1_ atom binds to three oxygen atoms of the Si–O framework and two oxygen atoms of reactant, making Rh_1_ exhibit positive to 0.927 |e|.

Isotope experiments suggest two necessary steps: activation of C–H bond of CH_4_ to form CH_3_ and insertion of CO to form acetic acid. Based on these experimental findings, reaction pathway on the Rh_1_O_5_ with lowest energy was simulated and transition states were located (Fig. 6). The energy profile and catalytic cycle are illustrated in Fig. 6b, c, respectively. The specific energies are listed in Supplementary Table 3. We found that the Rh_1_O_5_ active site (Fig. 6a) participates in the reaction by first activating C–H bond of methane (c2 and c3) with an activation barrier of 1.29 eV. It forms a methyl and hydroxyl adsorbed on the Rh atom (c4). Then, a CO molecule can insert to the Rh–O bond of Rh–O–H, forming a COOH adsorbed on Rh (c6). Then COOH can couple with the adsorbed methyl with a barrier of 1.11 eV (c7), forming a weakly adsorbed acetic acid (c8). Subsequent desorption yields the first CH_3_COOH molecule. The remaining Rh-O oxo group (c9) activates C–H bond of the second CH_4_ molecule to form a methyl and a hydroxyl group adsorbed on the Rh atom (c12). Following, or concurrently to this step, the second CO molecule binds to the unsaturated Rh site (c13). Then, the adsorbed CO inserts into the methyl–Rh bond with a barrier of 1.54 eV (c14), forming an acetyl group (c15). Finally, the hydroxyl group couples with carbon atom of C=O of the acetyl group to form the second acetic acid with a barrier of 0.72 eV (c17). Desorption of the second acetic acid molecule recovers the Rh site (c18), which then  bonds with a molecular O_2_, forming a Rh_1_O_5_ site (c1) ready for next catalytic cycle.

Our experimental studies show that high pressure of CO (Fig. 3b) in fact decreased the activity for producing acetic acid and finally poisoned the active sites. Computational study explored the observed influence of CO pressure on the catalytic activity. It suggests that saturated coordination of Rh with CO molecules under CO gas at a high pressure can poison a Rh_1_ site and thus prevent it from forming acetic acid. In addition, the DFT calculations show the activation energy for C*–*H of CH_4_ is largely increased if the Rh_1_ pre-adsorbed two CO molecules at high-pressure of CO (Supplementary Fig. 10b). More information can be found in Supplementary Discussion.

In summary, the heterogeneous catalyst, 0.10 wt%Rh/ZSM-5 consisting of singly dispersed Rh_1_O_5_ sites anchored in the micropores of microporous aluminate silicate was prepared. The anchored Rh_1_O_5_ sites exhibit unprecedented catalytic activity in synthesis of acetic acid higher than free Rh^3+^ in aqueous solution by >1000 times under mild conditions. This heterogeneous catalytic process opens a new route to synthesize acetic acid through direct utilization of methane under a mild condition at 150 °C or lower  by using a low-cost oxidant, O_2_ or air instead of current industrial process of synthesizing acetic acid through carbonylation of methanol.

## Methods

### Preparation and characterization of catalyst

Two steps were involved in the preparation of a Rh/ZSM-5 catalyst. The first step is the preparation of H-ZSM-5 by calcining zeolite NH_4_-ZSM-5 with a SiO_2_/Al_2_O_3_ ratio of 23 (Alfa Aesar) in air at 400 °C for 12 h. Four Rh/ZSM-5 catalysts with different Rh concentrations (0.01 wt%, 0.05 wt%, 0.10 wt%, 0.50 wt%) were synthesized through a method integrating vacuum pumping and IWI of aqueous solution containing certain amount of rhodium(III) nitrate hydrate (~36% Rh basis, Sigma-Aldrich) at room temperature. Typically, 500 mg of H-ZSM-5 was placed in a 50 mL three-port flask. The three ports were sealed with three corks. One port was connected to a vacuum pump. Before injection of Rh(NO_3_)_3_ solution, air in the flask containing 100 mg H-ZSM-5 was purged for 3–5 h by a vacuum pump when the H-ZSM-5 powder was being stirred. The size of stirring bar is5 mm for maximizing the amount of H-ZSM-5 to be stirred. Then, 0.30 mL of 1.0 mg/mL Rh(NO_3_)_3_ aqueous solution was added to the H-ZSM-5, which had been pumped for 3–5 h. The injection needle quickly reached the powder to avoid the dispersion of solution to the wall of flask since the environment of flask is in vacuum. In addition, the tip of needle was buried in the middle of H-ZSM-5 powder during injection, minimizing diffusion of solution to the wall of flask. During the injection, the H-ZSM-5 should be continuously stirred.

After the introduction of Rh^3+^, the samples were further dried in an oven at 80 °C for 3 h and calcined in air at 550 °C for 3 h. Supplementary Fig. 1 schematically shows the evolution of the structure of the anchored Rh atoms in ZSM-5 of 0.10 wt%Rh/ZSM-5. Actual Rh contents were determined by inductively coupled plasma atomic emission spectrometry (ICP-AES). TEM (FEI, Titan 80–300) was used to characterize the morphology of the catalyst. EXAFS of Rh K-edge was taken at SPring-8. For EXASF studies, the used catalyst of 0.10 wt%Rh/ZSM-5 was measured when the catalyst was kept at 150 °C in the flow of pure He. The adsorption fine structure spectra of Rh K-edge were fitted using IFEFFIT package and FEFF6 theory. Reference samples including Rh metal foil and Rh_2_O_3_ nanoparticles supported on Al_2_O_3_ were studied with EXAFS. Their r-space spectra of these reference samples were fitted with the same software. XPS was performed using a PHI5000 VersaProbe Spectrometer with monochromated Al Kα as X-ray source.

### Catalytic reactions

Transformation of methane to acetic acid on 0.10 wt%Rh/ZSM-5 was performed in a Parr high-pressure reactor (Series 4790, Parr) containing a Teflon liner vessel (Supplementary Fig. 3b). 28 milligram 0.10 wt%Rh/ZSM-5 was added to 10 mL H_2_O in the reactor. After evacuating the air left in reactor by flowing CH_4_ (99.9%, Matheson) and purging for five times, the system was pressurized with reactant gases in a sequence of CH_4_, CO (99.9%, Matheson) and O_2_ (99.9%, Matheson) to their desired pressures. The high-pressure reactor was completely sealed and then heated to the desired reaction temperature (typically 150 °C) by placing it in an oil bath. The temperature controller of the heating plate (VWR International) was used to measure the temperature of solution in the Parr reactor through the thermocouple placed in solution of Parr reactor and to control the temperature through outputting tunable power to the heating plate. Once the desired catalysis temperature was reached, the solution was vigorously stirred at 1200 rpm and was maintained at the reaction temperature for certain amount of time. After completion of the reaction, the vessel was cooled in an ice bath to a temperature below 10 °C to minimize the loss of volatile products. The solution with liquid products was filtered from the catalyst powder. The clean liquid containing acetic acid, formic acid, and methanol was analyzed by ^1^H-NMR or ^13^C-NMR. The concentration of Rh in the filtered powder was examined with ICP-AES as described in Supplementary Methods. Supplementary Fig. 11 is the standard curve of ICP-AES studies of Rh concentrations.

### Measurements of products with NMR and GC

^1^H NMR spectra were collected at room temperature on a Bruker AVANCE III HD 400 spectrometer at University of Notre Dame and University of Kansas. The measurements were calibrated by using 3-(trimethylsilyl)-1-propanesulfonic acid sodium salt (DSS) residual signal at *δ* = 0.0 ppm. Supplementary Fig. 4 is a typically NMR spectrum of products formed from CH_4_ transformation. Obviously, the peak of DSS can be identified. Typically, 0.7 mL collected filtrate and 0.1 mL of D_2_O (with 0.02 wt% DSS) were mixed in an NMR tube for analysis. The identified oxygenated products were acetic acid (*δ* = 2.08 ppm), formic acid (*δ* = 8.24 ppm) and methanol (*δ* = 3.34 ppm). A solvent suppression program was applied in order to minimize the signal originating from H_2_O, similar to our previous studies^11^. To quantify the products, standard curves were built using the same method as that of our previous report^11^. To establish a standard curve of a specific product such as acetic acid, a series of standard solutions with different concentrations of acetic acid were prepared. For instance, to establish a standard curve acetic acid, a series of standard solutions with different concentrations of acetic acid were prepared. NMR spectra of these standard solutions were collected with the exactly same parameters of NMR measurements. The ratio of the area of peak of acetic acid (*δ* = 2.08 ppm) to area of DSS of the same solution were calculated. These ratios of solutions with different concentrations of acetic acid were plotted as a function of the concentrations of acetic acid. This graph is a standard curve of acetic acid (Supplementary Fig. 5a), formic acid (Supplementary Fig. 5b), and methanol (Supplementary Fig. 5c). Concentration of a product (such as acetic acid) in a solution after catalysis in Parr reactor was determined by locating the ratio of the peak area of the product to the area of DSS on the *y*-axis of the standard curve (such as Supplementary Fig. 5a) and then finding the corresponding value on *x*-axis, which is the amount of the product of the solution after a catalysis in the unit of μmol. Gases in the head of Parr reactor after catalysis were analyzed with GC. Supplementary Table 4 presents the amounts of all reactants before catalysis and all products and left reactants after the catalysis; this catalysis was performed on 28 mg 0.10 wt%Rh/ZSM-5 dispersed in 10 mL deionized H_2_O under 50 bar CH_4_, 10 bar CO, and 8 bar O_2_ for  3 h. Bruker Topspin 3.5 software was used to acquire, process, and visualize the data.

### Data availability

All data are available from the authors upon reasonable request

## Electronic supplementary material

Supplementary Information
