# Supplementary material for: Single rhodium atoms anchored in micropores for efficient transformation of methane under mild conditions
Source: Nat Commun. 2018 Mar 26;9:1231. doi: 10.1038/s41467-018-03235-7 (PMC5964318; doi:10.1038/s41467-018-03235-7)
Supplement: Supplementary file 1 — Supplementary Information [file 41467_2018_3235_MOESM1_ESM.pdf]

Single rhodium atoms anchored in micropores for efficient transformation of methane under mild condition

Tang et al.

Supplementary Methods

ICP-AES measurements of concentrations of Rh in catalysts. ICP-AES was used in the measurements of Rh concentration in catalysts before and after catalysis. Four standard solutions with different concentration of Rh^{3+} (0.1ppm, 1ppm, 5ppm, 10ppm) were prepared by dissolving $Rh(NO_3)_3$ into de-ionized water. The volume of each solution is 40 ml. The standard curve was built through measuring the four solutions under the exactly same setup and parameters of the ICP-AES (mode: JY 2000 2 manufacture: HORIBA) and then plotting the known concentrations of the four solutions as a function of the optical emission spectrometry intensity. Supplementary Figure 11 is the plot of the known concentration of the solution as a function of the atomic emission spectrometry intensity. It is the standard curve for the measurements of concentration of Rh in the fresh and used catalysts.

To prepare a test solution of ICP-AES analysis, certain amount of fresh or used catalyst (0.10wt%Rh/ZSM-5) was dissolved in NaOH solution through simply mixing the accurately weighed catalyst into 10mL 1M NaOH solution and then sonicating the mixture for about 1 hr. Then, aqua regia (mixture of nitric acid and hydrochloric acid) was added to the solution until the pH was less than 5. The transparent solution was diluted by adding DI water to make the volume of the diluted solution to 30 mL. All test solution was tested under the under the exactly same setup and parameters of the same ICP-AES.

Isotope-labelled experiments using $^{13}CH_3OH$. To test whether acetic acid could form from carbonylaiton of CH_3OH on our catalyst 0.10wt%Rh/ZSM-5, 1.0 mmol isotope-labeled $^{13}CH_3OH$ (99 atom% ^{13}C, Aldrich) was added to 10 ml deionized H_2O before introduction of 10 bar CH_4, 5 bar CO, and 4 bar O_2 to the Parr reactor. The purpose of adding isotope-labeled $^{13}CH_3OH$ to H_2O before catalysis is to test whether $^{13}CH_3OH$ could act as an intermediate to react with CO on the catalyst to form isotope-labelled acetic acid, $^{13}CH_3COOH$. In reaction pathway β, acetic acid is formed through carboxylation of methanol (Figure 5a); methanol should be involved definitely. The reaction pathway α in Figure 5a does not involve CH_3OH. Figs. 5b presents the three sets of possible products if $^{13}CH_3OH$ was added as a probe agent. Figure 5c and 5d are a regular ^1H NMR spectrum of solution obtained from 10 bar CH_4, 5 bar CO and 4 bar O_2 and the ^1H NMR spectrum of solution obtained from 10 bar CH_4, 5 bar CO and 4 bar O_2 with added 1 mmol $^{13}CH_3OH$, respectively.

Calculations of TOR of 0.10wt%Rh/ZSM-5. Catalytic activities of these catalysts reported in literatures[1,2] and the Rh_1O_5@ZSM-5 of this work were calculated in terms of the number of product molecules per Rh site per second. They are listed in Table 1 of the main text. The following paragraphs will describe how they were calculated.

For our catalyst, Rh_1O_5@ZMS-5 (0.10wt%Rh/ZSM-5), as shown in Figure 2, 846 μmol of acetic acid was formed from 28 mg of catalyst at 150°C for 12 hrs under the mixture of mixture of 50 bar CH_4, 10 bar CO, and 8 bar O_2; the concentration of rhodium in the catalyst is 0.10wt%. The amount of all Rh atoms is $\frac{28\times10^{-3} gram \times 0.10\%}{104\, gram\ per\ mol\ Rh} = 2.8 \times 10^{-7} mol$. By assuming all Rh atoms anchored to ZSM-5 participate into this catalysis, TOR for production of acetic acid can be calculated as the following:

$TOR = \frac{845\times10^{-6}\ mol\times N_A}{2.8\times10^{-7}\times N_A\times12\times3600\ second} = 0.070$ acetic molecules per Rh site per second. This TOR was listed in entry 2 of Table 1.

With the same calculation method of TOR, TORs of acetic acid and organic oxygentates under catalysis condition 1 (mixture of 50 bar CH_4, 10 bar CO and 8 bar O_2 for 2 hrs) were calculated with the yields of acetic acid and organic oxygentates presented in Figure 2; these TORs were listed in entry 1 of Table 1.

Calculations of TOR of Rh cations without any support in aqueous solution. A similar experiment was performed. 5 ml of 0.01 mol/l $Rh(NO_3)_3$ was added in Parr reactor and then 50 bar CH_4, 10 bar CO and 8 bar O_2 was introduced the Parr reactor. The reaction was performed at 150°C for about 90 hrs. With the same calculation method, the TORs were calculated. The TORs of all organic products (CH_3COOH, CH_3OH and HCOOH) and TOR of acetic acid are 2.4×10^{-5} organic molecules and 6.3×10^{-6} acetic acid molecules per Rh site per second generated from homogeneous catalyst $Rh(NO_3)_3$ without any promoter. They are listed in entry 3 of Table 1.

Methods of DFT calculations. The periodic density functional theory (DFT) calculations were performed using the Vienna ab initio Simulation Package (VASP).[3,4] The Perdew-Burke-Ernzerhof (PBE)[5] functional of generalized-gradient approximation (GGA) was used for the electron exchange and correlation. The D3 method for van der Waals correction by Grimme is used. [6] The electron-core interaction was described using the projector-augmented wave method (PAW).[7,8] The kinetic energy cutoff was set to 450 eV for the plane wave basis set, and the Brillouin zone was sampled using the gamma point only. A section of a relaxed ZSM-5 framework was used in a cluster model and the dangling bonds capped with hydrogen. The ZSM-5 cluster was placed in a 18×18×22 $Å^3$ box. The Rh site and first neighbors were allowed to relax during the subsequent calculations with the rest of the cluster fixed. The adsorption energies were calculated using $E_{ads} = E_{cluster+adsorbate} - (E_{cluster} + E_{adsorbate})$, where the energy of the adsorbate $E_{adsorbate}$ was computed by placing the adsorbate in a 15 Å wide cubic cell. Transition states were found using the climbing image nudged elastic band method implemented in VASP, using eight images and a force convergence criterion of 0.05 eV $Å^{-1}$. [9]

Supplementary Note

Ready separation of products from solvent by using dodecane. Transformation of CH_4, CO and O_2 to organic oxygenates was performed at 150°C for 2 hrs on 28 mg of catalyst while solvent dodecane was used. The yields of acetic acid and formic acid in dodecane under a mixture of 30 bar CH_4, 10 bar CO and 5 bar O_2 at 150°C for 4 hrs are 225 μmol and 82 μmol, respectively (Supplementary Figure 8). The advantage of using dodecane is the ready separation of hydrophilic product molecules from hydrophobic solvent molecules.

Supplementary Discussion

Does acetic acid form from reaction of CO with formic acid? To test whether acetic acid could form through reaction between formic acid and CO, we performed three experiments by adding

20 mg 0.10wt%Rh/ZSM-5 into 10 ml H_2O, dispersing HCOOH into 10 ml DI H_2O and introducing 0 bar CO, 5 bar CO or 10 bar CO and then heating the solution to 150°C and remaining it at 150°C for 3 hrs. As shown in Supplementary Figure 7, there was no any acetic acid formed in the experiments. Thus, formation of acetic acid from coupling between formic acid and CO is not a possible pathway for synthesis of acetic acid from CH_4, CO and O_2.

Does acetic acid form from dry reforming of CH_4? Direct reforming CH_4 with CO_2 to produce acetic acid at a temperature ≥250°C was reported in literatures.[10-14] Presumably, one potential reaction pathway for the formation of acetic acid on our catalyst is that CO could be first oxidized by O_2 to form CO_2 and then CO_2 could couple with CH_4 to form acetic acid. To check this possibility, 30 bar CH_4 and 30 bar CO_2 were introduced to the Parr reactor and the reaction was performed under the same catalytic condition on 28 mg 0.10wt%Rh/ZSM-5 (at 150°C for 5 hrs). As shown in Supplementary Figure 6d, no acetic acid, formic acid or methanol was formed. Thus, the pathway consisting of CO oxidation to from CO_2 and then reforming CH_4 with CO_2 to form acetic acid was excluded.

Preservation of Rh cations in micropores after catalysis. One concern is whether Rh cations were still in the micropores after catalysis. Solution after catalysis consisted of solvent and products (in liquid) and solid catalyst. As most zeolite particles deposited to the bottom, they were readily separated after centrifugation. Notably, small particles couldn't be precipitated; thus, we used filter paper to filter these small catalyst particles from the solution after majority catalyst particles were deposited through centrifugation. In this way, the most solid catalyst particles were collected for ICP analysis.

The collected catalyst (after catalysis) was dissolved in solution for ICP test. The details of preparation solution were described in the section entitled "ICP-AES measurements of concentrations of Rh in catalysts" of Supplementary Methods. ICP-AES tests showed that the Rh atoms in the collected catalyst was 0.098wt%Rh, very close to the original weight ratio of Rh, 0.10wt%Rh. It suggested that there was little leaching of Rh from ZSM-5. From this point of view, Rh cations remained in the micropores during catalysis.

Does Rh cations chemically bond to O atoms in micropores? A fundamental question is whether Rh cations chemically bond to oxygen atoms in micropores or only physisorb in the micropores. To check the oxidation state and coordination environment of the Rh atoms in micropore after catalysis, XANES and EXAFS studies of the used catalysts were performed. The measured distance between Rh and O atoms from r-space of Rh K-edge (Figure 1e) is 2.015 Å, which is very close to the Rh-O bond length of Rh_2O_3 reference sample. Thus, Rh cations are definitely anchored on oxygen atoms of micorpores. In addition, the observation of peaks α, Rh-(O)-Aland β, Rh-(O)-Si in r-space spectrum of Rh K-edge of 0.10wt%Rh/ZSM-5 after catalysis (Figure 1e) further supported that Rh atoms anchor on oxygen atoms of the wall of the micropores of ZSM-5.

Why selectivity for producing formic acid is higher at a shorter reaction time? The catalytic performances in Figure 2 obtained at 10 bar CH_4, 10 CO and 8 bar O_2 for 2 hrs and 50 bar CH_4, 10 CO and 8 bar O_2 for 2 hrs in Figure 2 and data in Figure 3 were catalysis data collected after 1.5 or 2 hrs. The selectivity for formation of formic acid is higher than that for acetic acid. However, a longer reaction time such as the data under the catalytic conditions (in the mixture of

10 bar CH_4 with 10 bra CO and 8 bar O_2 for 12 hrs or the mixture of in 50 bar CH_4 with 10 bar CO and 8 bar O_2 for 12 hrs in Figure 2 gave selectivity for formation of acetic acid higher than formic acid.

The high selectivity for formic acid (the low selectivity for acetic acid) is relevant to the large portion of incubation heating of catalyst from 25°C to 150°C among a whole heating when the formal heating time at 150°C is short. Here the whole heating of catalyst includes the incubation heating from 25°C to ideal temperature (typically 150°C) and formal heating at the ideal temperature (typically 150°C); the time reported for heating is only the time of reactor remaining at ideal temperature (typically 150°C); the time used for heating the catalyst from 25°Cto 150°C (called incubation heating) is about 1 hr. If the formal heating time at ideal temperature is only 2 hrs or even 1 hr, the incubation heating is be an important portion of the whole heating. If the formal heating time at ideal temperature is 12 hrs, the incubation heating is a minor portion of the overall heating.

Since the selectivity for formation of formic acid in heating of short time is higher than that of heating of long time, it suggests that a relatively low temperature of incubation heating from 25°C-150°C favors the formation of formic acid. When the heating time is only 1 or 2 hrs, the incubation heating from 25°C to 150°C probably mainly forms formic acid and thus results in a relatively high selectivity for the formation of formic acid. This interpretation is consistent with the proposed reaction pathway by DFT calculation. As shown in the energy profile Figure 6b, to form acetic acid, the barrier to across the transition state (c7 in Figure 6c) from c6 to c8 in Figure 6c to form the first acetic acid is quite high. This high barrier makes the formation of the first acetic acid at low temperature not kinetically favorable. Alternatively, the intermediate (c6 in Figure 6c), a formate (HCOO) adsorbed in Rh could readily couple with one H to form formic acid at low temperature to desorb from the site, instead of crossing the high barrier of the transition state (c7 in Figure 6) to form acetic acid.

To further check whether this interpretation is correct or not, we performed time-dependent study of the yields of formic acid and acetic acid, respectively. The parallel studies were done for formal heating at 150°C for 0.5 hrs, 2 hrs, 3hrs, 5, hrs and 12 hrs under the same condition (50 bar CH_4 10 bar CO and 8 bar O_2); as plotted in Supplementary Figure 9, the selectivity for formation of acetic acid increases as a function of time. This is consistent with the kinetically favorable formation of formic acid at low temperature since an experiment with short formal heating time at 150°C has a large portion of heating at low temperature (25°C-150°C). Thus, our interpretation of the high selectivity for producing formic acid is supported by the experiments in Supplementary Figure 9.

Understand the CO pressure-dependent catalytic activity through computation. Our experimental studies found that CO at high pressure (Figure 3b) in fact decreased the selectivity for producing acetic acid and finally poisoned the active sites. To understand this observation, we evaluated the CO adsorption on the Rh_1 atom in DFT calculations. We found that the first adsorbed CO molecule binds strongly to the Rh_1 site, with an adsorption energy of -1.92 eV (Supplementary Figure 10a), and -0.69 eV for the second CO (Supplementary Figure 10b). It suggests that the Rh_1 site could adsorb two CO molecules.

We also explored the C-H activation of methane by Rh_1 atom when the Rh_1 has already adsorbed a CO molecule (Supplementary Figure 10c). Supplementary Figure 10c is the transition state in activation of the first C-H of CH_4 on Rh_1 with one pre-adsorbed CO molecule. With a pre-adsorbed CO molecule on Rh_1O_5, the barrier for activating the first C-H of CH_4 is only 0.34 eV.

Unfortunately, the activation barrier for activating CH_4 on a Rh_1 atom with two pre-adsorbed CO molecules is increased to 1.36 eV. The large increase of barrier for activating CH_4 suggested by DFT calculation rationalized the poison of CO to Rh_1O_5 sites in the formation of acetic acid when CO pressure is higher than 10 bar, observed in Figure 3b.

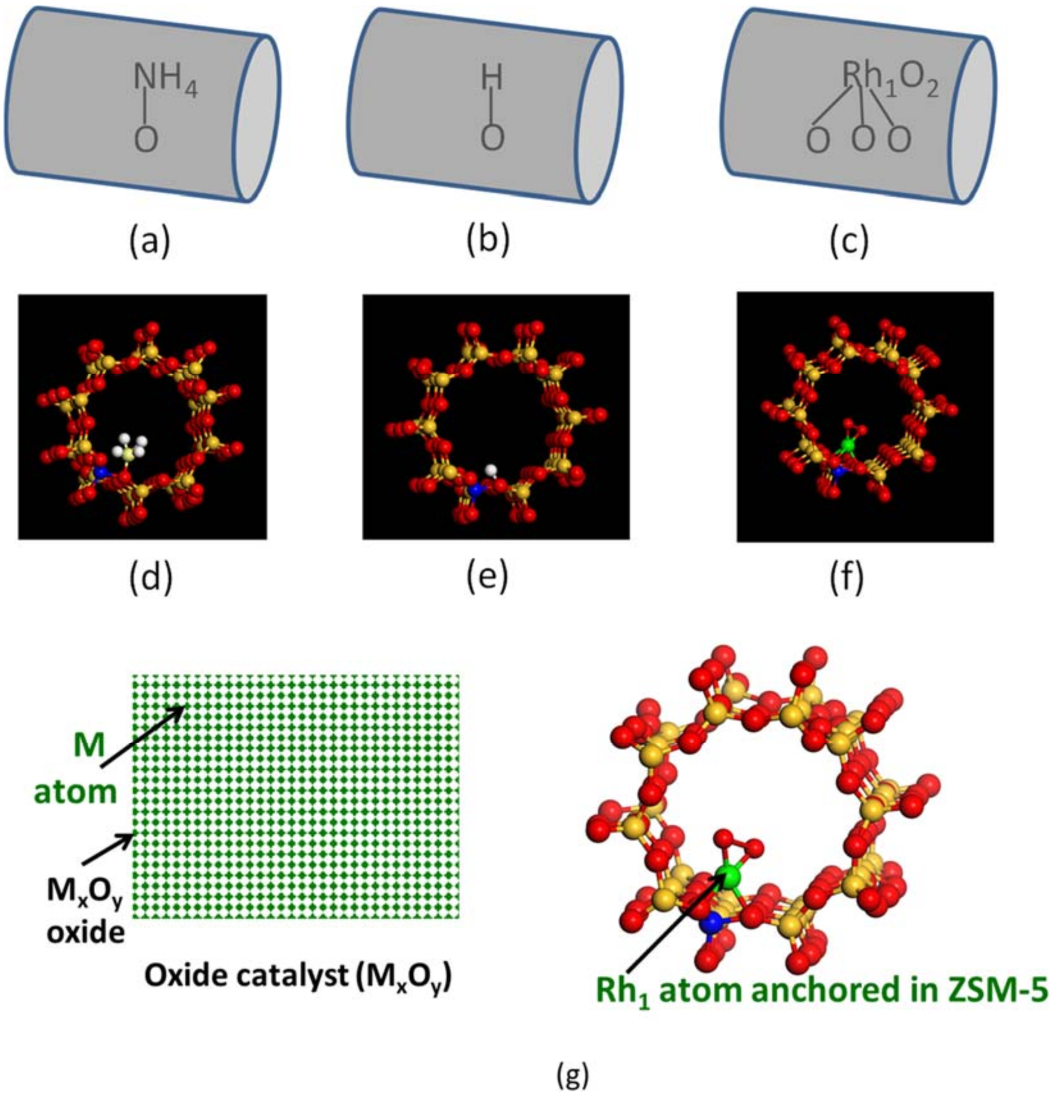

Supplementary Figure 1. Schematic showing synthesis and structural feature of isolated Rh atoms in ZSM-5. Schematic (a-c) and pictures (d-f) showing the preparation of 0.10wt%Rh/ZSM-5 catalyst in which Rh_1O_5 sites were formed in the micropores of aluminosilicate. (a) NH_4-ZSM-5 as pruchased. (b) Formation of H-ZSM-5 upon calcination of NH_4-ZSM-5 at 450ºC for 3 hrs. (c) Formation of ZSM-5 with anchored Rh_1O_5 sites through intgrated vacuum pumping and incipident wetness impregenation (IWI) of Rh^{3+} to pores through ion-exhange in aqueous soltuion; after ion exhange, catlayst precurosr was centrifuged; the preciptatant was dried in an oven at 80ºC for 3 h and calcined in air at 550ºC for 3 h. (d), (e), and (f) are ball-stick model figures of the corresponding schematics (a), (b), and (c). (g) Schematic showing the difference between continuously packed cationic sites on M_xO_y oxide nanoparticles and separately anchored Rh cations in ZSM-5.

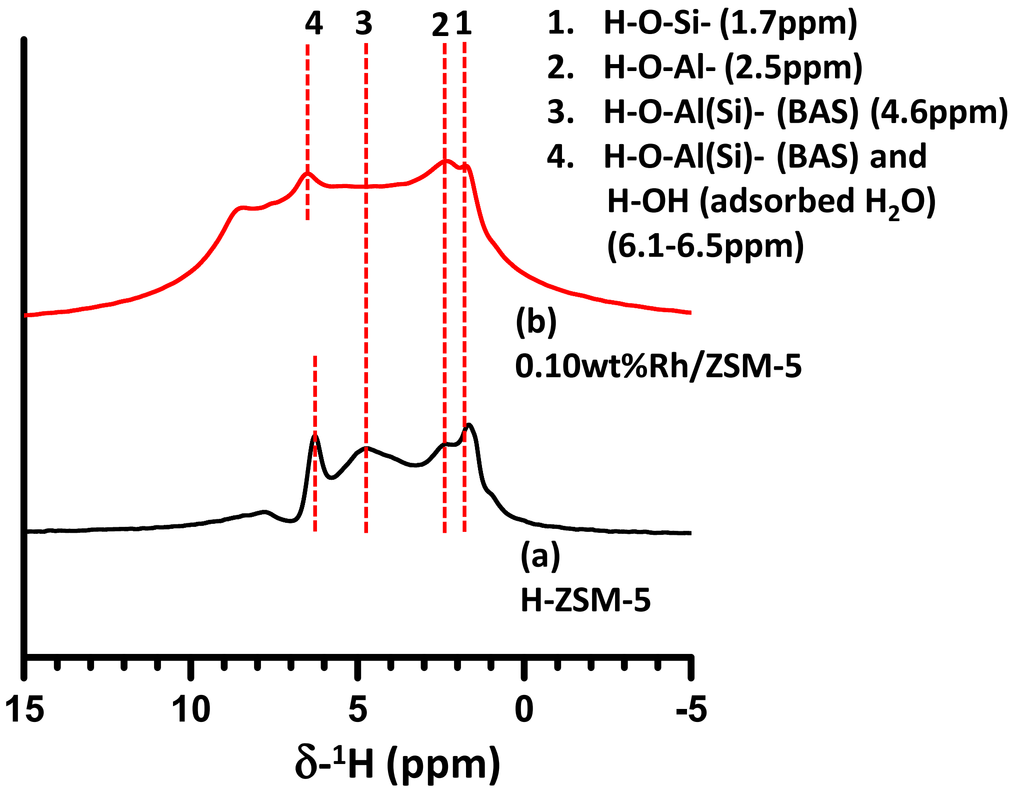

Supplementary Figure 2. ^{1}H MAS NMR for H-ZSM-5 (a) and 0.10wt%Rh/ZSM-5 (b). The peak at 4.6 ppm was obviously observed in H-ZSM-5 but not clear in 0.10wt%Rh/ZSM-5. The peak at 4.6 ppm was assigned to Brønsted acid sites (BAS) based on the values reported in literature.[11] This difference shows that some Brønsted sites of H-ZSM-5 lost due to the replacement by Rh cations in the ion exchange (IWI process). Peak at 1.7 ppm was assigned to the H-O-Si- (on external frame of ZSM-5) based on the literature.[11]. The peak at 6.1 ppm is contributed from adsorbed H_2O molecules based on the literature[11] and from the second type of Brønsted acid sites (BAS) based on literature[16]. The references of these assignments were presented in Table S1. To remove the contribution of H_2O to ^{1}H spectra, catalyst was loaded to a stainless tube reactor and then were annealed to 400ºC and remained for 5 hrs while they were being pumped by a vacuum pump. After 5 hrs annealing at 400ºC in vacuum to remove H_2O and other impurity, the valves at the two sides of the stainless tube were closed. The stainless steel tube containing the dry catalyst was transferred to a glove box. A similar treatment for removal of water molecules adsorbed in ZSM-5 was used in literature.[15] Then, the valve of the stainless steel tube was open to transfer the sample to a NMR test tube. The NMR test tube was then sealed. The sealed NMR test tube was immediately used for NMR studies.

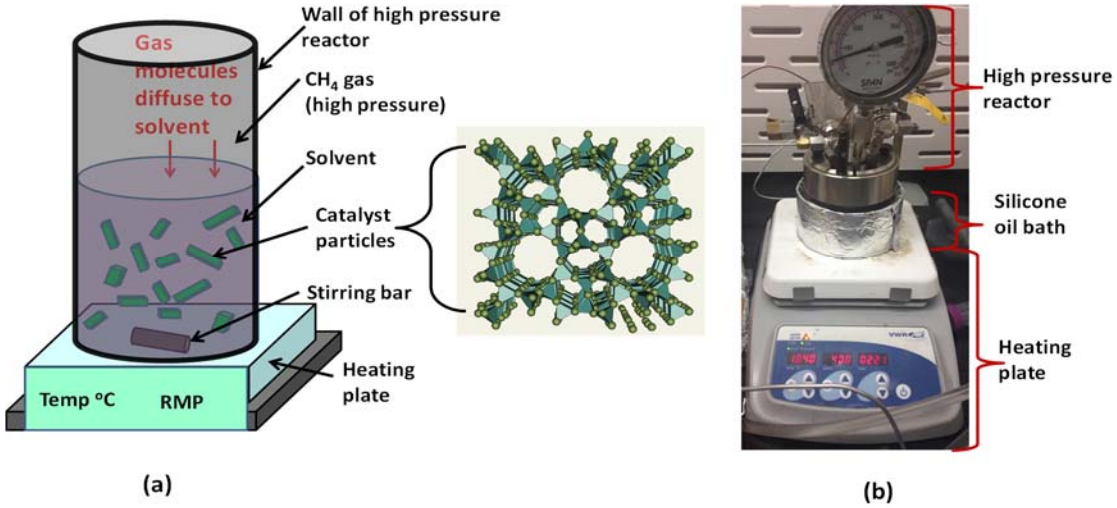

Supplementary Figure 3. Setup of the catalytic transformation of CH4 to acetic acid and other products in a Parr reactor. Typically, 28 mg catalyst was loaded to the catalyst and dispersed in 10 ml (aqueous water or dodecane). Gases with certain pressure was introduced and mixed. The Parr reactor containing catalyst and mixture of reactants was loaded to an oil bath. Output power of the heating plate was modulated by the K-type thermocouple insert to the liquid solution in the Parr reactor. The reading temperature during catalysis is T±1°C; here T is typically 150ºC. When temperature of the liquid in the Parr reactor reaches the set temperature such as 150ºC, stirring bar started to stir to make the solid catalyst dispersed in liquid homogeneously.

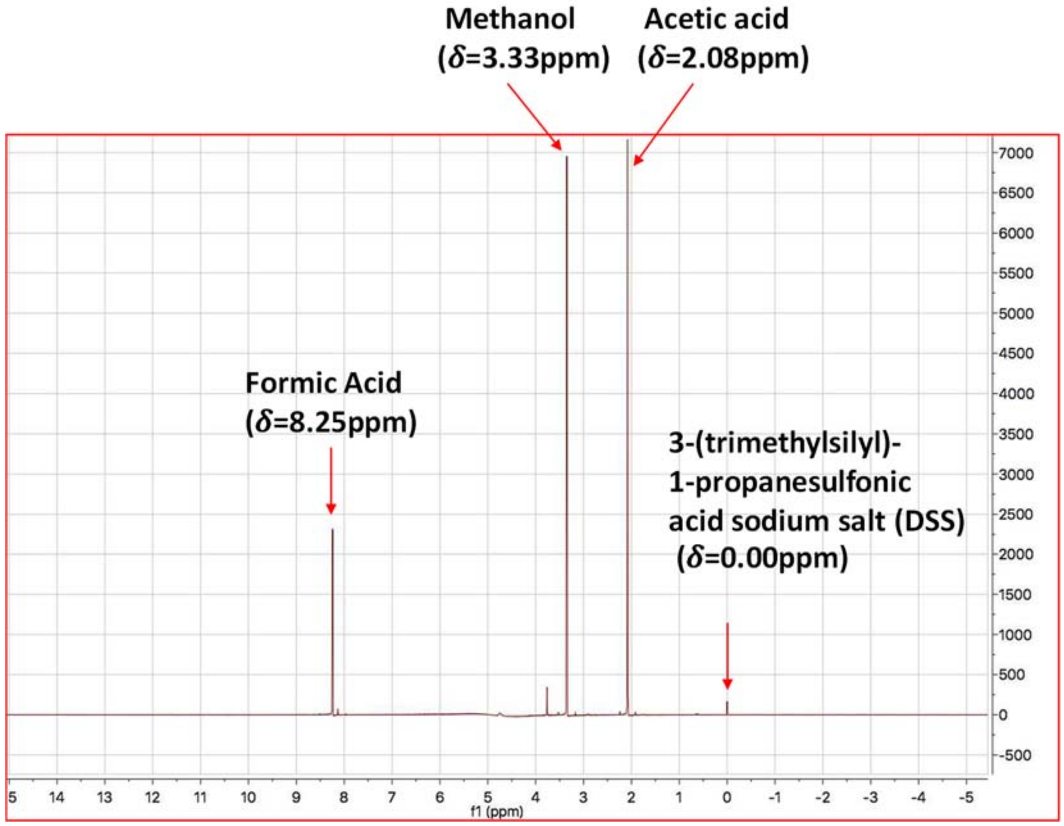

Supplementary Figure 4. ^{1}H-NMR spectrum of solution in Parr reactor after chemical transformation of CH_4 to acetic acid, methanol and formic acid on 0.10wt%Rh/ZSM-5.

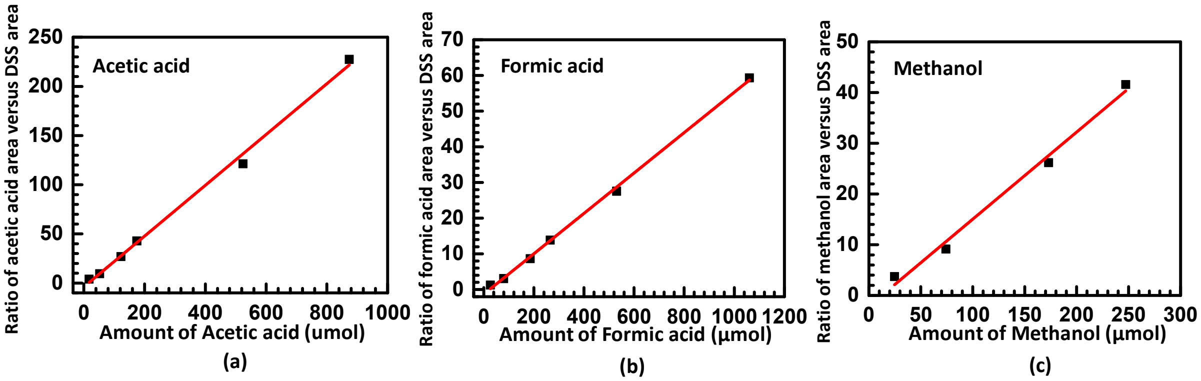

Supplementary Figure 5. Standard curves built for measurements of amount of acetic acid (a), formic acid (b), and methanol (c) through ^{1}H-NMR spectra.

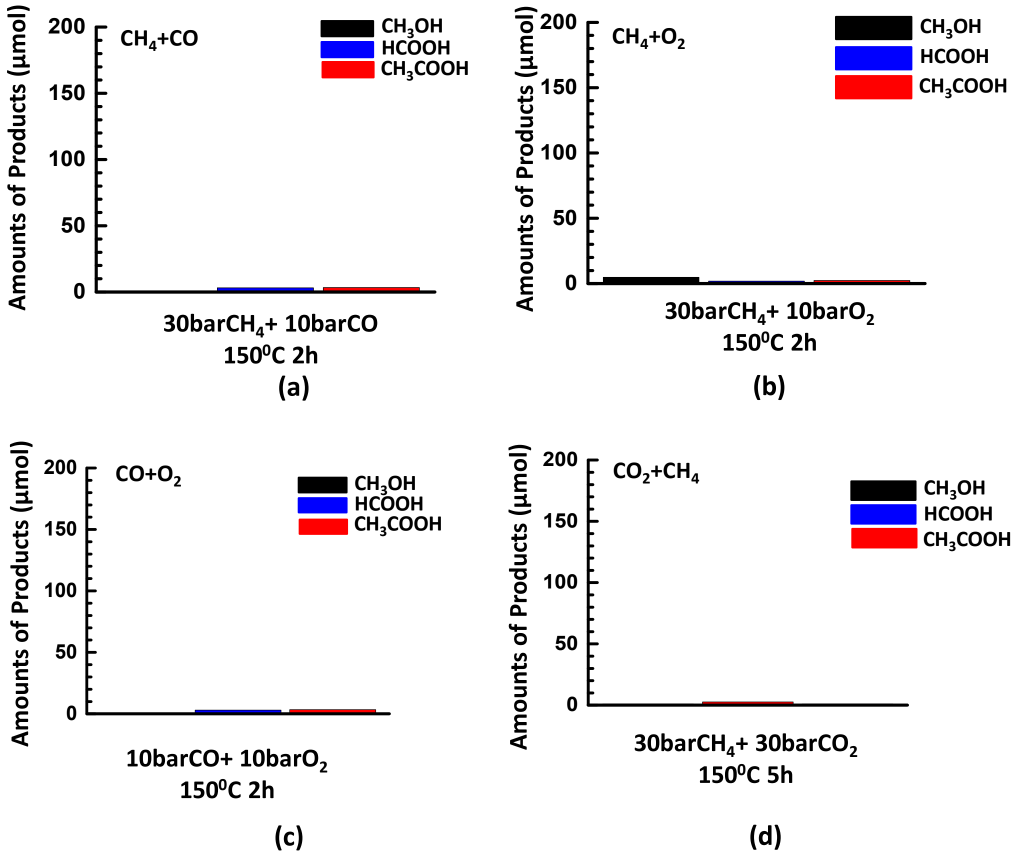

Supplementary Figure 6. Yields of acetic acid, formic acid and methanol from 28 mg 0.10wt%Rh/ZSM-5 in aqueous solution at 150°C for certain amount of time as shown in figures in (a) the mixture of 30 bar CH_4 and 10 bar CO, (b) the mixture of 30 bar CH_4 and 10 bar O_2, (c) the mixture of 10 bar CO and 10 bar O_2, and (d) the mixture of 30 bar CO_2 and 30 bar CH_4.

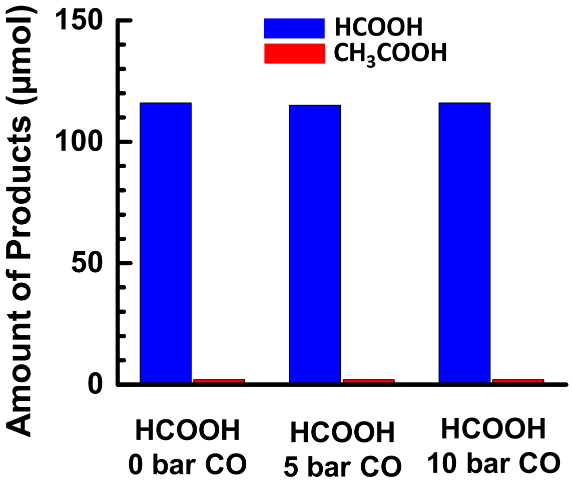

Supplementary Figure 7. Yields of acetic acid when 118 μmol HCOOH was mixed with different pressure of CO at 150ºC for 3 hrs.

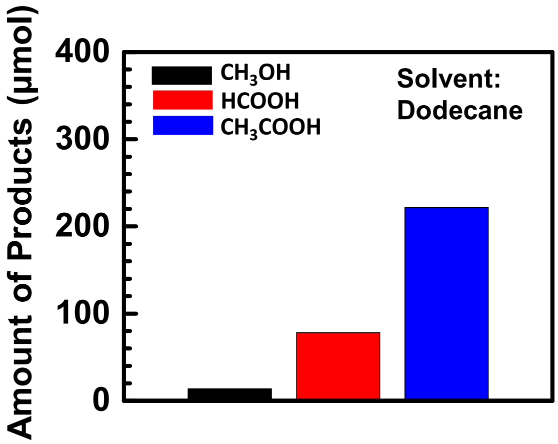

Supplementary Figure 8. Yields of acetic acid, formic acid and methanol from 28 mg 0.10wt%Rh/ZSM5 in 10 ml dodecane at 150ºC in the mixture of 30 bar CH_4, 10 bar CO and 5 bar O_2 for 4 hrs.

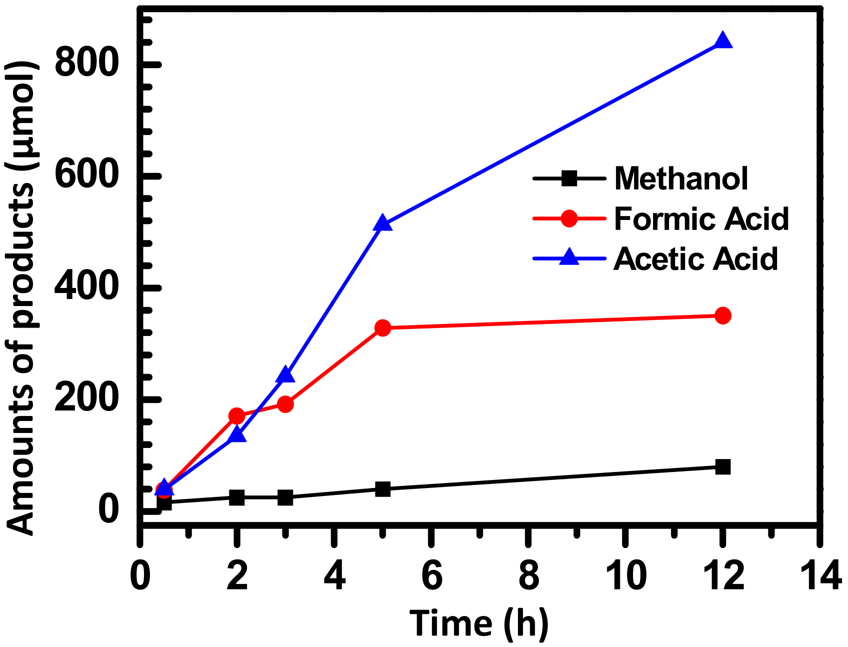

Supplementary Figure 9. Evolutions of yields of acetic acid, formic acid, and methanol as a function of reaction time under a condition of 50 bar CH_4, 10 bar CO, and 8 bar O_2 at 150ºC. Five experiments were performed under the same catalytic condition (50 bar CH_4, 10 bar CO, and 8 bar O_2 at 150ºC) with 28 mg 0.10wt%Rh/ZSM-5; the difference among these five parallel experiments is their reaction time (0.5, 2, 3, 5, and 12 hrs).

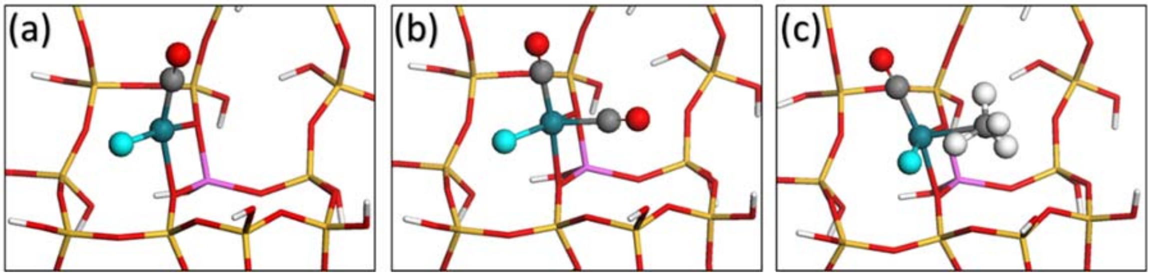

Supplementary Figure 10. Structures generated from calculation studies in exploring the effect of pressure of CO gas on the Rh active site. (a) The adsorption configuration of CO on the Rh_1 site binding to one CO is show for one CO. (b) The adsorption configuration of CO on the Rh_1 binding to two CO molecules. (c) The transition state of C-H activation of CH_4 on Rh_1O_5 binding to one CO molecule.

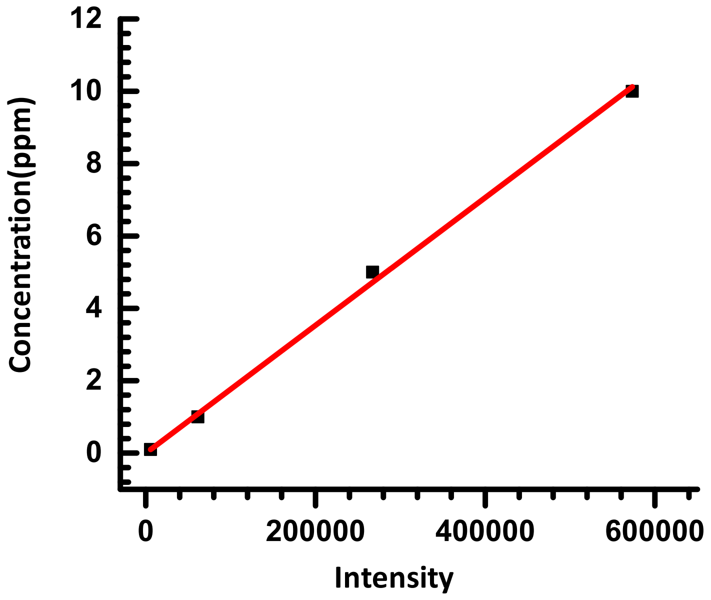

Supplementary Figure 11. Standard curves for measurement of concentration of Rh cations in solution with ICP-AES.

Supplementary Table 1. Assignment for ^{1}H NMR spectra of H-ZSM-5-based catalysts.

Group	Chemical shift(ppm)	References
-Si-OH	1.8	
-Al-OH	2.6	Reference[11]
BAS(-Si-OH-Al)	4.0	
-Si-OH	2.2	
-Al-OH	2.9	Reference [15]
BAS(-Si-OH-Al)	4.4	
-Si-OH (Z74)	2.4	
-Al-OH (Z74)	3.3	
Type-1 BAS(-Si-OH-Al) (Z74)	4.3	Reference[16]
Type-2 BAS(-Si-OH-Al) (Z74)	6.5	
H_2O adsorbed (Si/Al=74)	6.9	
H_2O adsorbed (Si/Al=180)	6.3	

Supplementary Table 2. Catalytic performances of 28 mg catalysts with different loading of rhodium dispersed in 10 ml H_2O under gas phase of a mixture of 10 bar CH_4, 5 bar CO and 2 bar O_2 at 150°C in a Parr reactor. The reaction time is 1 hr.

Entry	Catalyst	Mixture of reactants (bar)	Temperature (°C)	Methanol (µmol)	Formic acid (µmol)	Acetic acid (µmol)	Total products (µmol)
1	H-ZMS-5	CH_4: 10 bar<br>CO: 5 bar<br>O_2: 2bar	150	3.7±5.0	2.3±5.0	1.9±5.0	7.8±5.0
2	0.01wt%Rh/ZSM-5	CH_4: 10 bar<br>CO: 5 bar<br>O_2: 2bar	150	7.4±5.0	4.6±5.0	5.7±5.0	17.6±15.0
3	0.05wt%Rh/ZSM-5	CH_4: 10 bar<br>CO: 5 bar<br>O_2: 2bar	150	12.4±5.0	89.4±5.0	44.0±5.0	145.8±15.0
4	0.10wt%Rh/ZSM-5	CH_4: 10 bar<br>CO: 5 bar<br>O_2: 2bar	150	14.0±5.0	153.4±5.0	58.7±5.0	246.0±15.0
5	0.50wt%Rh/ZSM-5	CH_4: 10 bar<br>CO: 5 bar<br>O_2: 2bar	150	11.2±5.0	144.1±5.0	86.6±5.0	241.8±15.0

Supplementary Table 3. Energies and activation barriers for the simulated pathway leading to the formation of acetic acid as shown in Figure 6 of the main text.

**Reaction Step**	**Energy (eV)**	**Barrier (eV)**
Rh	0.00	
RhO_2	-1.61	
RhO_2-CH_4	-1.94	
RhO_2-CH_4--TS	-0.65	1.29
RhO_2-CH_3-H	-2.04	
RhO-CH_3-OH	-3.10	
RhO-CH_3-COOH	-4.69	
RhO-CH_3-COOH--TS	-3.58	1.11
RhO-CH_3COOH	-5.26	
RhO	-4.32	
RhO-CH_4	-5.06	
RhO-CH_4--TS	-4.04	1.02
RhOH-CH_3	-5.68	
RhOH-CH_3-CO	-8.25	
RhOH-CH_3-CO--TS	-6.71	1.54
RhOH-CH_3CO	-7.56	
RhOH-CH_3CO--TS	-6.84	0.72
Rh-CH_3COOH	-7.13	
Rh	-5.64	

Supplementary Table 4. Analysis of carbon-containing reactants before catalytic transformation of CH_4, CO and O_2 and the left carbon-containing reactants and the formed carbon-containing products in Parr reactor.

	Carbon of CH_4 (μmol)	Carbon of acetic acid (μmol)	Carbon of formic acid (μmol)	Carbon of methanol (μmol)	Carbon of CO (μmol)	Carbon of CO_2 (μmol)	Carbon in total (μmol)
Before catalysis	20848.4				3207.4		24055.8
After catalysis	19857.2	224.2	146.3	37.0	2046.0	None	22534.9

Note: catalytic condition: 28 mg 0.10wt%Rh/ZSM-5 in 10 ml deionized H_2O, 50 bar CH_4, 10 bar CO and 8 bar O_2, 3 hrs of reaction time.

Supplementary References

1. Periana, R. A., Mironov, O., Taube, D., Bhalla, G. & Jones, C. J. Catalytic, oxidative condensation of CH4 to CH3COOH in one step via CH activation. *Science* **301**, 814-818, (2003).
2. Lin, M. & Sen, A Direct Catalytic Conversion of Methane to Acetic-Acid in Aqueous-Medium, *Nature* **368**, 613-615 (1994).
3. Kresse, G. & Furthmuller, J. Efficiency of ab-initio total energy calculations for metals and semiconductors using a plane-wave basis set. *Comp. Mat. Sci.* **6**, 15-50 (1996).
4. Kresse, G. & Furthmuller, J. Efficient iterative schemes for ab initio total-energy calculations using a plane-wave basis set. *Phys. Rev. B* **54**, 11169-11186 (1996).
5. Perdew, J. P., Burke, K. & Ernzerhof, M. Generalized gradient approximation made simple. *Phys. Rev. Lett.* **77**, 3865 (1996).
6. Grimme, S., Antony, J., Ehrlich, S. & Krieg, H. A consistent and accurate ab initio parametrization of density functional dispersion correction (DFT-D) for the 94 elements H-Pu. *J. Chem. Phys.* **132**, 154104 (2010).
7. Kresse, G. & Joubert, D. From ultrasoft pseudopotentials to the projector augmented-wave method. *Phys. Rev. B* **59**, 1758 (1999).
8. Blöchl, P. E. Projector augmented-wave method. *Phys. Rev. B* **50**, 17953-17979 (1994).
9. Henkelman, G., Uberuaga, B. P. & Jónsson, H. A climbing image nudged elastic band method for finding saddle points and minimum energy paths. *J. Chem. Phys.* **113**, 9901-9904 (2000).
10. Lustemberg, P. G. *et al.* Room-Temperature Activation of Methane and Dry Re-forming with CO2 on Ni-CeO2(111) Surfaces: Effect of Ce3+ Sites and Metal-Support Interactions on C-H Bond Cleavage. *ACS Catal.* **6**, 8184-8191 (2016).
11. Wu, J. F. *et al.* Mechanistic Insight into the Formation of Acetic Acid from the Direct Conversion of Methane and Carbon Dioxide on Zinc-Modified H-ZSM-5 Zeolite. *J. Am. Chem. Soc.* **135**, 13567-13573 (2013).
12. Huang, W. *et al.* Possibility of direct conversion of CH4 and CO2 to high-value products. *J. Catal.* **201**, 100-104 (2001).
13. Zhang, R. G., Song, L. Z., Liu, H. Y. & Wang, B. J. The interaction mechanism of CO2 with CH3 and H on Cu (111) surface in synthesis of acetic acid from CH4/CO2: A DFT study. *Appl. Catal. A: Gen.* **443**, 50-58 (2012).
14. Yahi, N., Menad, S. & Rodriguez-Ramos, I. Dry reforming of methane over Ni/CeO2 catalysts prepared by three different methods. *Green Process Synth.* **4**, 479-486 (2015).
15. Yu, Z. W. *et al.* Bronsted/Lewis Acid Synergy in H-ZSM-5 and H-MOR Zeolites Studied by H-1 and Al-27 DQ-MAS Solid-State NMR Spectroscopy. *J. Phys. Chem. C* **115**, 22320-22327 (2011).
16. Heeribout, L., Doremieux-Morin, C., Nogier, J. P., Vincent, R. & Fraissard, J. Study of high-silica H-ZSM-5 acidity by H-1 NMR techniques using water as base. *Microporous Mesoporous Mater.* **24**, 101-112 (1998).
